# Competition for hosts modulates vast antigenic diversity to generate persistent strain structure in *Plasmodium falciparum*

**DOI:** 10.1371/journal.pbio.3000336

**Published:** 2019-06-24

**Authors:** Shai Pilosof, Qixin He, Kathryn E. Tiedje, Shazia Ruybal-Pesántez, Karen P. Day, Mercedes Pascual

**Affiliations:** 1 Department of Ecology and Evolution, University of Chicago, Chicago, Illinois, United States of America; 2 School of BioSciences, Bio21 Institute/University of Melbourne, Melbourne, Australia; 3 Santa Fe Institute, Santa Fe, New Mexico, United States of America; University of Colorado, Boulder, UNITED STATES

## Abstract

In their competition for hosts, parasites with antigens that are novel to the host immune system will be at a competitive advantage. The resulting frequency-dependent selection can structure parasite populations into strains of limited genetic overlap. For the causative agent of malaria, *Plasmodium falciparum*, the high recombination rates and associated vast diversity of its highly antigenic and multicopy *var* genes preclude such clear clustering in endemic regions. This undermines the definition of strains as specific, temporally persisting gene variant combinations. We use temporal multilayer networks to analyze the genetic similarity of parasites in both simulated data and in an extensively and longitudinally sampled population in Ghana. When viewed over time, populations are structured into modules (i.e., groups) of parasite genomes whose *var* gene combinations are more similar within than between the modules and whose persistence is much longer than that of the individual genomes that compose them. Comparison to neutral models that retain parasite population dynamics but lack competition reveals that the selection imposed by host immunity promotes the persistence of these modules. The modular structure is, in turn, associated with a slower acquisition of immunity by individual hosts. Modules thus represent dynamically generated niches in host immune space, which can be interpreted as strains. Negative frequency-dependent selection therefore shapes the organization of the *var* diversity into parasite genomes, leaving a persistence signature over ecological time scales. Multilayer networks extend the scope of phylodynamics analyses by allowing quantification of temporal genetic structure in organisms that generate variation via recombination or other non-bifurcating processes. A strain structure similar to the one described here should apply to other pathogens with large antigenic spaces that evolve via recombination. For malaria, the temporal modular structure should enable the formulation of tractable epidemiological models that account for parasite antigenic diversity and its influence on intervention outcomes.

## Abstract 2

A combination of computational modeling and empirical data reveals persistent strain structure despite vast antigenic diversity in the human malaria parasite Plasmodium falciparum, with potential consequences for the acquisition of immunity.

## Introduction

The dynamic arms race between hosts and pathogens sets the stage for a selective advantage of rare variants that confer either immune protection to hosts or immune escape to pathogens and a corresponding disadvantage of common ones. This frequency-dependent effect can act as a form of balancing selection and is a powerful force promoting high antigenic diversity and maintaining polymorphisms significantly longer than neutral drift. High diversity and temporal persistence of diversity at the gene level have been shown in numerous host-pathogen systems. Genes that encode pathogen resistance in hosts, such as the major histocompatibility complex [1], and those that underlie antigenic variation in parasites, such as the *var* multigene complex in the malaria parasite *P*. *falciparum*, display exceptional polymorphism compared to other functional genes. Several components of *var* genes are known to have originated millions of years ago and to be shared with closely related species that infect apes [2].

In transmission systems, diversity and persistence are also relevant at a higher level of organization than that of individual genes. In particular, pathogen strains concern temporally persistent combinations of genes related to infection, including those encoding antigens. The role of immune selection at this higher level of organization remains, however, poorly understood and documented, especially in pathogens whose antigenic variation involves vast diversity generated via recombination within the genome or between different genomes, as is the case in several bacteria, protozoa, and fungi [3]. Can strains exist and persist in such vast antigenic spaces? What is a strain in dynamic systems undergoing recombination at the level of both the genes themselves and the genomes they compose? A key characteristic signature of immune selection would involve the persistence of gene combinations over longer time scales than expected under neutrality [4], a hypothesis that remains to be examined despite its relevance for the existence and definition of strains themselves.

We address this temporal dimension by examining the role frequency dependence plays in maintaining gene combinations over time in the highly diverse multicopy *var* gene family of *P*. *falciparum*. Frequency-dependent selection in pathogen systems is analogous to stabilizing competition in ecological communities [5, 6]. Competition can drive coexisting species to self-organize into clusters with limiting similarity along a niche or trait axis [7–10]. In epidemiology, strain theory had proposed that competition for hosts through cross-immunity, the cross-protection conferred by previous acquisition of immunity, can structure pathogen populations into temporally stable sets of genetically distinct strains with limited overlap of antigenic repertoires [11]. Theoretical studies for low-to-medium genetic diversity predicted parasite strains with no or limited genetic overlap [12, 13], including in the case of multicopy genes [14]. For *P*. *falciparum*, recent work allowing for realistic levels of genetic diversity comparable to that found in endemic high transmission regions of sub-Saharan Africa resulted in a more complex similarity structure clearly distinguishable from patterns generated under neutrality but that can no longer be described by distinct clusters [15]. Deep sampling and sequencing of *var* gene isolates from asymptomatic human populations within a given time window or transmission season have confirmed these nonrandom patterns that are also non-neutral, in the sense that they cannot be simply explained by stochastic extinction, immigration, and transmission in the absence of acquired immune memory and therefore competition of parasites for hosts [15–17]. The lack of explicit consideration of the temporal dimension in the analysis of structure, despite the highly dynamic nature of the *P*. *falciparum* system, may be the reason why no apparent distinct clustering was identified, and it is the motivation behind this work.

Analyzing the temporal dimension requires simultaneously tracking the population dynamics of genomes and their genetic relationship in a hyperdiverse system. An additional challenge is allowing for evolution via recombination. Here, we apply multilayer networks to characterize patterns of genetic similarity in *var* gene repertoires through time for both a theoretical model and an empirical data set from Ghana, unique in its depth of sampling and coverage over multiple seasons. The patterns of modularity we describe should be relevant to other pathogen systems, whether possessing multicopy gene families [3] or multiple independent loci encoding different antigens.

## Results

The *var* genes encode the major antigen of the blood stage of infection, *P*. *falciparum* erythrocyte membrane protein 1 (PfEMP1) [18]. Besides immune evasion, PfEMP1 promotes adherence of erythrocytes to blood vessels, leading to disease manifestations. Hence, *var* genes play an important role in malaria epidemiology. Each parasite genome has a “repertoire” of 50–60 unique *var* gene copies sequentially expressed to produce different variants of PfEMP1 during an infection. In endemic regions of high transmission, *var* genes exhibit enormous diversity [16, 17, 19], resulting from evolutionary innovation at two levels of organization. At the gene level, *var* gene variants can be generated through both mutation and ectopic recombination [20–23], with tens of thousands of variants documented in local populations [16]. At the repertoire level, variation in gene composition is obtained through sexual (meiotic) recombination in the mosquito vector [20, 24, 25].

**Fig 1:**
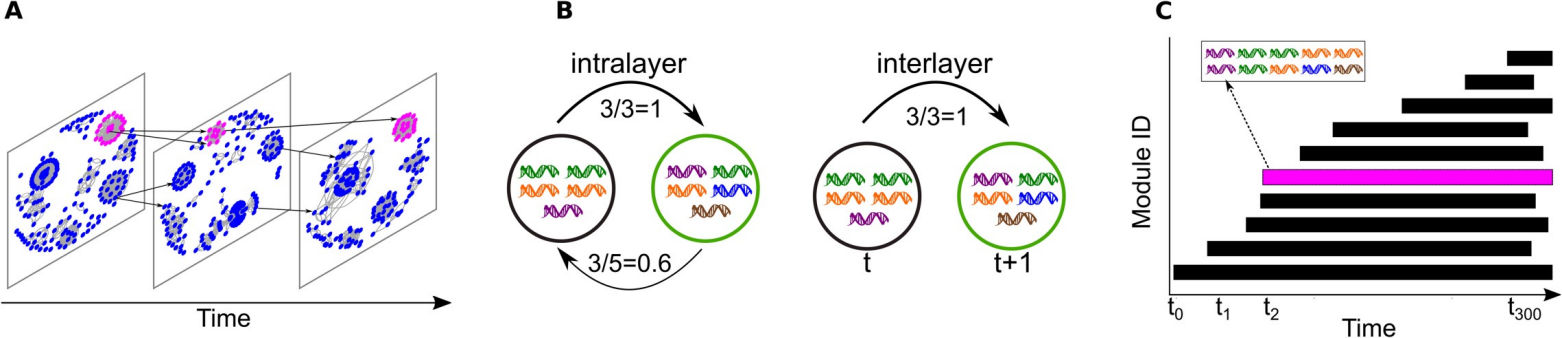
A toy example for a temporal multilayer network of repertoire genetic similarity and its associated modular structure. In (A), each layer represents a time point. The network in each time point depicts the genetic similarity between pairs of repertoires, where each repertoire is a combination of alleles (see Methods for the reason why we used alleles instead of *var* genes). The measure of genetic similarity is asymmetric to take into account the asymmetric competition resulting from different numbers of distinct allele variants, as shown in the example in (B): the two repertoires share 3 alleles, but the black one has 3 unique alleles and the green has 5, and so the later will outcompete the former (alleles are depicted as DNA symbols with different colors). Interlayer edges between repertoires leading from any time point *t* to time point *t* + 1 were defined in the same way, but these edges only point in one direction to represent temporal flow. For clarity, we present only a few interlayer edges in (A). (C) We used an algorithm for community detection in networks to identify “modules” (depicted as rectangles), which contain repertoires that are more genetically similar to each other than to repertoires from other modules. Modules persist in time, but the number and identity of the repertoires that compose them can change (for example, repertoires can be removed by host immunity and can appear as a result of recombination). New modules appear in time, while others die out. Pink repertoires in (A) are part of the same module illustrated by the same color in (C).

Transmission and *var* diversity are positively correlated in the evolutionary history of malaria; i.e., areas of low and high transmission have evolved towards low (for example, South America) and high (for example, sub-Saharan Africa) diversity, respectively. Because our questions target systems of high diversity and transmission, we focus on this regime in the main text and present results for low and medium diversity in S1 Text. We simulated populations using an agent-based model (ABM) and sampled them monthly for 25 years with a total of 300 time points, where each time point is a snapshot of changes accumulated in the parasite population during a 30-day period. We used a temporal multilayer network to analyze population structure in these time series. Competition between repertoires, as well as their evolution and persistence, are intimately interlinked processes emanating from genetic differences. Hence, each layer (time point) was constructed to represent a network of genetic similarity, in which nodes are repertoires and intralayer edges encode genetic similarity between repertoire pairs in terms of their *var* gene composition (Methods; [15]). Using the same index, we connected layers to each other via unidirectional interlayer edges depicting the genetic similarity between a repertoire in time *t* to those in time *t* + 1 (Fig 1; Methods). This definition of interlayer edges maps the genetic relationship among repertoires, which underlies competition—the process determining persistence—into the network, explicitly creating temporal dependency between layers. We characterized network structure by looking for modules using Infomap, an algorithm that explicitly considers the multilayer structure and the temporal “flow” associated with interlayer edges [26–28]. These modules (i) contain repertoires that are genetically more similar to each other than to other repertoires in the network and (ii) are defined within and across layers (across time). Under immune selection, two repertoires that are clustered to the same module can be thought of as sharing a similar niche in human immune space. I.e., they are able to infect hosts with more similar immune profiles than parasites from other modules.

### The temporal and non-neutral population structure

**Fig 2:**
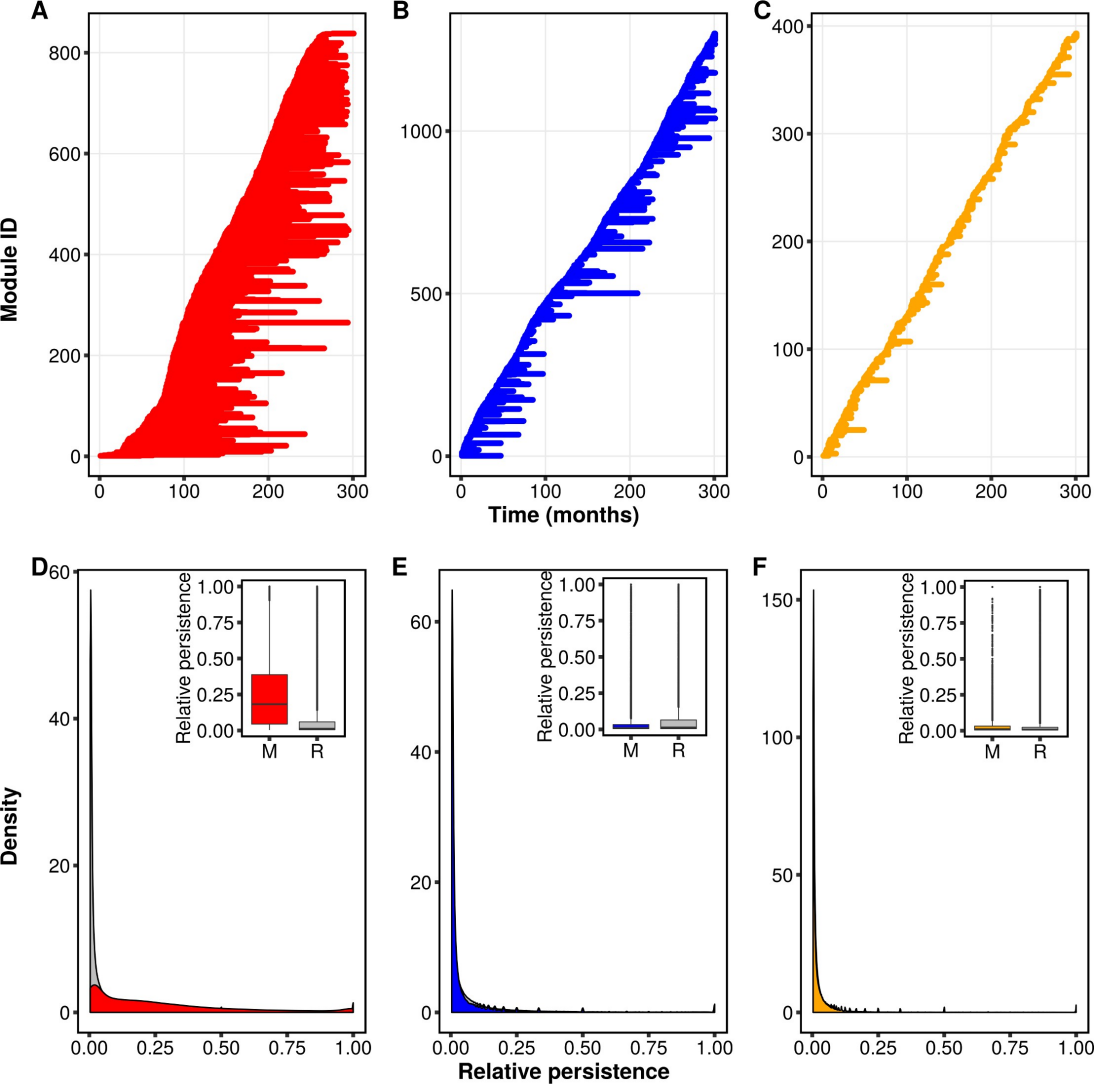
Temporal population structure in the high-diversity regime. The left, middle, and right columns represent the selection (red), generalized immunity (blue), and complete neutrality (orange) scenarios, respectively. (A,B,C) Example of population structure from one run of the ABM. Each line on the *y*-axis corresponds to a different module, and its horizontal length depicts its occurrence across layers. Modules are generated and die out. (D) The selection scenario is characterized by modules (red) that persist for much longer than the repertoires (gray) that compose them. The inset is a comparison between the values in the red and gray density plots using box plots. (E,F) In the neutral scenarios, modules and repertoires have similar relative persistence. See Methods for details on how we calculated relative persistence. Results in (D)–(E) are for data pooled across 50 runs of the ABM. Data from model output used to produce the figure can be found in https://figshare.com/articles/Figure_data/8079713. ABM, agent-based model; M, module; R, repertoire.

Under both immune selection and the two neutral scenarios, we find a dynamic modular structure, in which modules are constantly generated and die out. Interestingly, the structure generated in the presence of immune selection exhibits long-lived coexisting modules, whereas the one generated in the two neutral models is characterized by short-lived ones (Fig 2A, 2B and 2C). We can examine the role of immune selection by comparing the persistence of repertoires to that of modules. When immune selection is at play, we find that modules persist longer than the repertoires that compose them (Fig 2D). This mismatch in persistence indicates that modules are not necessarily composed of the same repertoires across time and that repertoires do not necessarily have to persist as long as the module they occupy. By contrast, in the neutral scenarios, modules are short-lived compared to their repertoires (Fig 2E and 2F). This contrast suggests that the process of module formation is inherently different between the scenarios: under neutrality, genetic changes accumulate due to antigenic drift until a point is reached at which the repertoire population is sufficiently different to create a new set of modules. Conversely, a modular structure under selection is a result of the stabilizing competition between repertoires, which pushes them to be as dissimilar as possible. Indeed, limiting similarity and module coexistence in the selection scenario leads to a more even distribution of the number of repertoires in modules compared to neutrality (S6 Fig). The uneven distribution of module sizes in the neutral scenarios is a result of (i) short-lived modules with low tendency to coexist and (ii) the high number of recombinant repertoires, which in the absence of immune selection are not purged and thus fill the antigenic space and blur the clear niche separation observed in the selection scenario. Taken together, these results indicate that immune selection acts as a form of balancing selection, maintaining long-lived modules.

### Comparison to empirical data

We tested our theoretical findings using parasite isolate data collected in an age-stratified longitudinal study in the Bongo District (BD), Ghana [29]. This area is characterized by high seasonality, with a prolonged dry season (November–May) and a short wet season (June–October). Our data set contains 6 surveys (Svs) carried out at the end of both the wet and dry seasons (EWS and EDS) (S1 Table); it is the only longitudinal data set available of an asymptomatic *P*. *falciparum* reservoir population in a given location using deep sequencing.

To validate our theoretical results with empirical data requires generating an expectation benchmark for the population structure under the empirical sampling scheme with the ABM. To this end, we first need to corroborate our theoretical predictions when the seasonality of the sampling area is included (see Methods for details). We find that, although seasonality does leave a signature in the formation of modules (S9 Fig), the differences between the immune selection and the neutral scenarios remain qualitatively similar (S10 Fig). Next, we ran 100 ABM benchmark simulations with parameter values that capture our uncertainty of the exact local parameters in the sampling area, as was done in [15]. Beyond seasonality, these benchmark simulations consider the specific characteristics of our empirical data: only 6 layers (as compared to 300 in the theoretical results) and three sessions of indoor residual spraying (IRS) conducted as interventions during the sampling period. Our goal is not to fit the benchmark simulations to the data, but rather to show that when we imitate the empirical data with our ABM, the immune selection scenario still produces modules with higher persistence than the two neutral scenarios and is also more similar to the empirical data.

**Fig 3:**
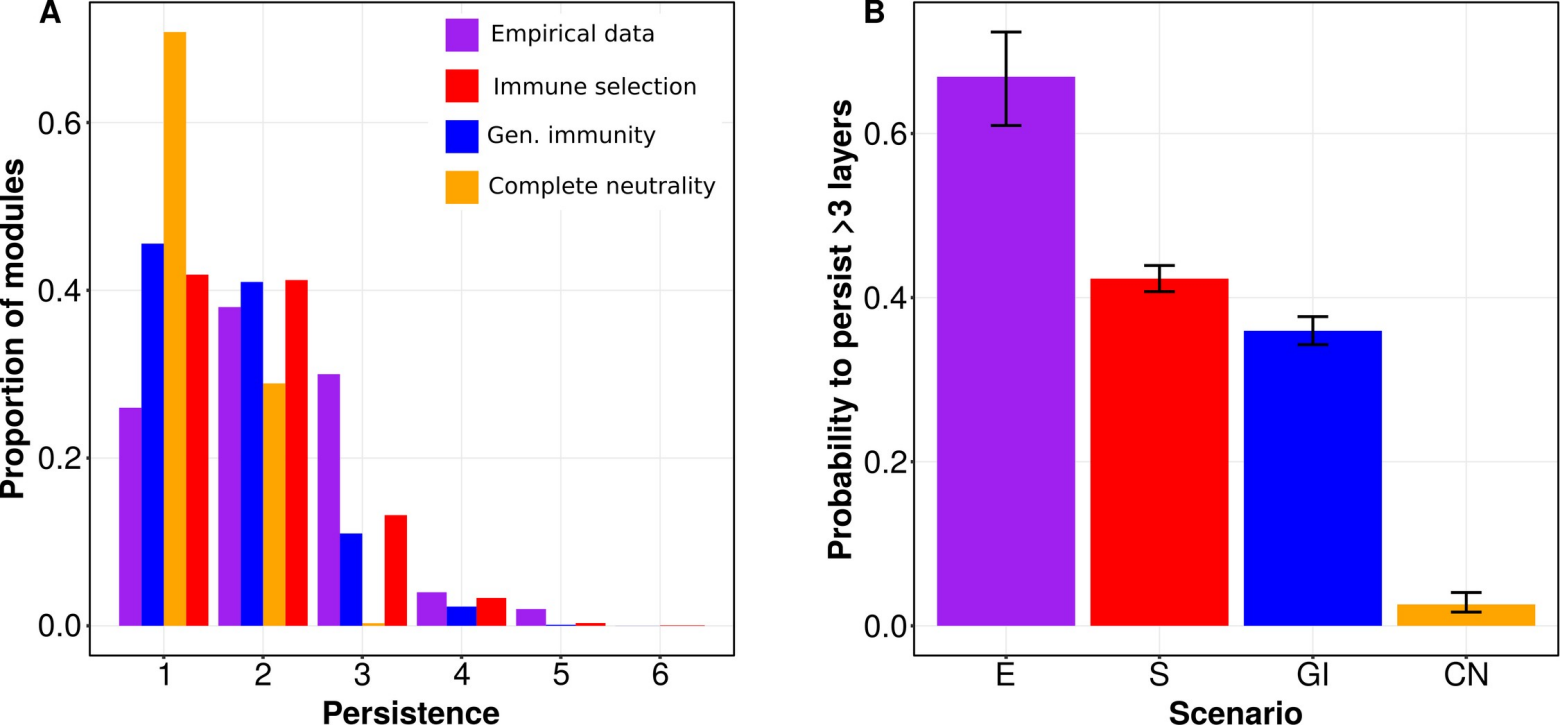
Comparison of empirical data to ABM simulations. (A) Proportion of modules that persisted for a given number of layers in the empirical data (purple) compared to ABM simulations of the selection (red), generalized immunity (blue), and complete neutrality (orange) scenarios. (B) Predicted probability (by logistic regression) that a module in each of the scenarios will persist for at least 3 layers. Note that we can only include modules that were born in layer 3 or before. All scenarios are different from each other, and these differences are statistically significant. In particular, there is a statistically significant difference between selection and generalized immunity (Wald test, *χ*^2^ = 28.1, *P* < 0.0001). Data from model output and empirical data used to produce the figure can be found in https://figshare.com/articles/Figure_data/8079713. ABM, agent-based model; CN, complete neutrality; E, empirical data; GI, generalized immunity; S, immune selection.

We find that module persistence is indeed longer under immune selection than under neutrality (Fig 3A). Results of logistic regression show that the probability that a module persists for at least 3 layers is higher under immune selection than under either of the two neutral scenarios and that this difference is statistically significant. This probability is also closer to that of the empirical data (Fig 3B; we chose here 3 layers because no module persisted for longer under complete neutrality). Because we matched by construction the distribution of infection duration in the generalized immunity model to that of the immune selection one (see Methods), module persistence probabilities are more similar to each other between these two scenarios than to those generated under complete neutrality (yet still significantly different; Wald test, *χ*^2^ = 28.1, *P* < 0.0001). Therefore, even with a short time window and one that includes an intervention, we are able to show differences consistent with those expected from our theoretical results. This analysis provides evidence for the role of immune selection in maintaining niche coexistence and persistence in an empirical setting.

### Epidemiological consequences of strain structure

The modular structure we have uncovered may affect epidemiological parameters because immunity gained to a repertoire from one module facilitates immunity to other repertoires from the same module, and infection with a repertoire from a different module adds exposure largely to alleles that have not been seen before. Also, despite modularity, genetic similarity between any two repertoires is generally low because of the very large diversity of the gene pool to begin with. Thus, to examine whether the modular structure does have an epidemiological meaning, we used duration of infection as a measure. Specifically, we generated curves describing the decline in infection duration with accumulated infections in an individual host. By sampling from the parasite population structure generated by the ABM under the three scenarios, we simulated the accumulation of infections in naive hosts for 1 year, explicitly incorporating the epidemiological force of infection of the system.

**Fig 4:**
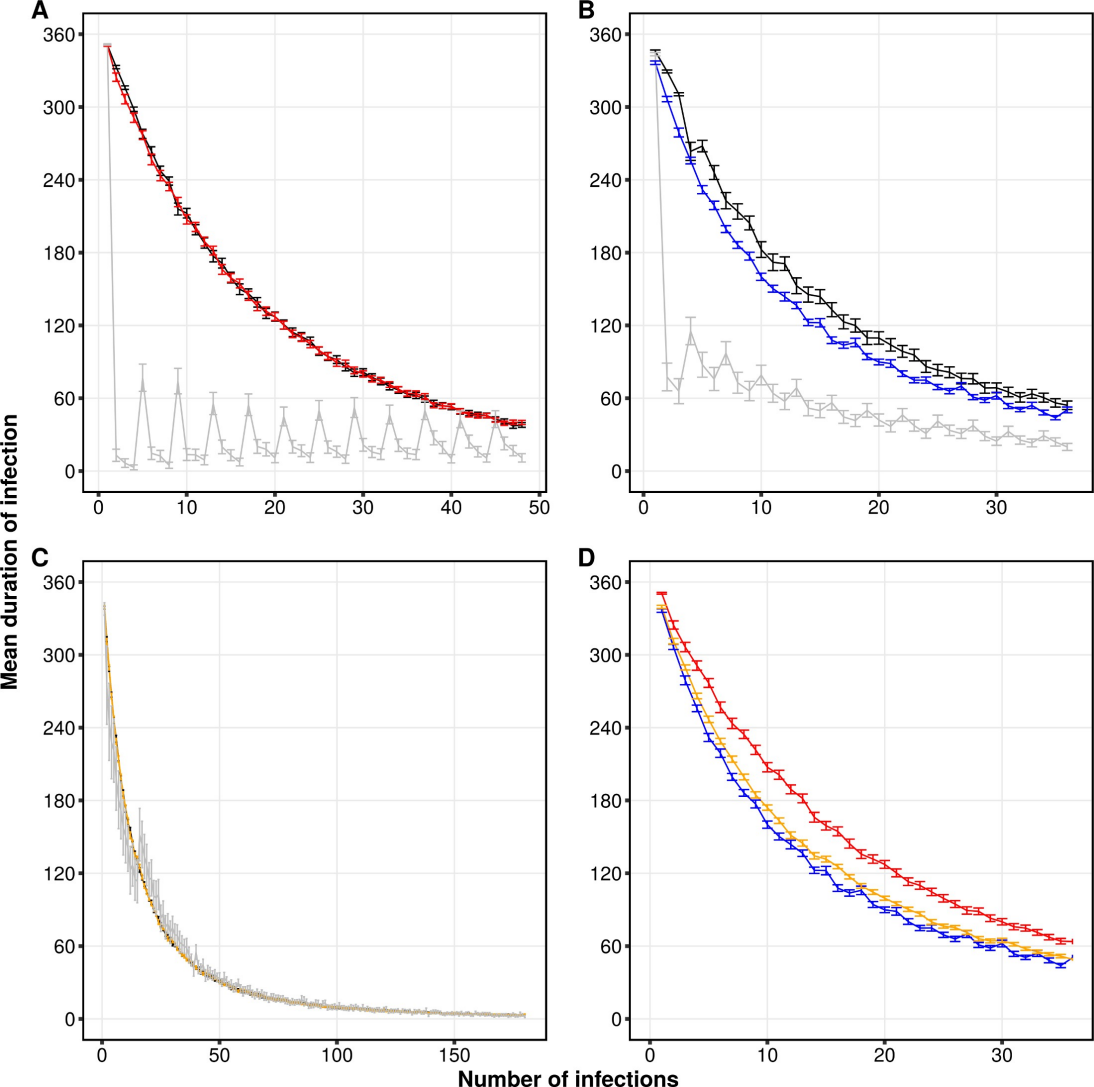
Epidemiological consequences of repertoire population structure under high *var* gene diversity. The figure shows the decline in duration of infection as a function of the number of infections accumulated in a naive host during a 1-year period. Panels (A), (B), and (C) correspond respectively to selection, generalized immunity, and complete neutrality. In any given simulation, a naive host was infected with repertoires originating either from the same module (within-module infections; gray), from different modules (between-module infections; black), or randomly (colored; red: immune selection, blue: generalized immunity; orange: complete neutrality). Each point is the average duration of infection across 50 runs of the ABM for 10 hosts with 5 random starting layers in 10 different modules (see Methods and S1 Text for details on experimental design). We are interested in the comparison of the decreasing trends for the duration of infection across scenarios. The small fluctuations overlaid on these trends reflect intermittent increases in this duration (they are the result of the way infections were sampled over discrete time steps, by advancing from one layer to the next, in the design of the simulations; see S1 Text for details). The number of infections in each layer was determined as a function of the EIR of the ABM. (D) A direct comparison of the curves for random infections from panels (A)–(C). The curve of immune selection is above that of the neutral scenarios because under immune selection, repertoires consist of a lower proportion of identical alleles than under neutrality. In all panels, error bars are 95% confidence intervals. Data from model output used to produce the figure can be found in https://figshare.com/articles/Figure_data/8079713. ABM, agent-based model; EIR, entomological inoculation rate.

We find that the duration of infection declines at a faster rate when hosts are infected with repertoires originating from the same module compared to infections with repertoires from different modules (Fig 4A). In generalized immunity, we still observe this difference between the curves, though to a lesser extent (Fig 4B). Under complete neutrality, however, population structure has no effect on duration of infection (Fig 4C) because modules are extremely short-lived, and the difference in genetic diversity between repertoires that belong to either the same or different modules is low. This analysis indicates that the modular population structure generated by immune selection can exhibit epidemiological consequences. It also suggests that modules can be viewed as “strains” that are epidemiologically different from each other from the perspective of host immunity.

With this result in mind, we then explored the epidemiological consequence of structure resulting from immune selection in a more realistic way. In nature, infections can occur with repertoires belonging or not to the same module. We therefore additionally sampled repertoires uniformly at random, regardless of their module affiliation (colored curves in Fig 4A, 4B and 4C). Under selection, this generates a curve that overlaps with that of the between-module infections because under high diversity, limiting similarity pushes modules to be as different as possible from each other. A direct comparison of these curves (Fig 4D) shows that in populations structured by immune selection, the duration of infection is generally higher than in populations assembled by stochastic processes alone (the red curve is above the other two). This is because competition drives repertoires to be as different from each other as possible, making the genetic diversity within a repertoire higher on average under immune selection.

An exponential fit of the form $d=ae^{-b_{i}}$ (where *d* is the duration of infection and *i* is the number of accumulated infections) to these curves for the first 50 infections indicates an approximately 28% and 15% faster decline in *d* under generalized immunity and complete neutrality, respectively, compared to immune selection (*b*_*selection*_ = 0.0502 ± 0.000159 SE; *b*_*generalized*_ = 0.0647 ± 0.000234 SE; *b*_*neutral*_ = 0.0577 ± 0.000129 SE). These model fits, which consider the entomological inoculation rate (EIR) of each of the scenarios, show a consistently slower reduction in duration of infection under selection than under neutrality. For example, the predicted duration of a new infection in a 5-month–old infant would be 163, 131, and 5 days under selection, generalized immunity, and complete neutrality, respectively (S13 Fig).

## Discussion

We find that competitive interactions between genomes for hosts act as a stabilizing force that dynamically organizes the hyperdiversity of *var* gene repertoires into temporally persistent modules. These clusters reflect dynamic “niches” emerging in the immune space of the human host population. By considering the temporal dimension, we therefore recover the clustered nature characteristic of stabilizing competition/balancing selection, which was not evident in static analyses at high gene diversity [15]. This clustering is also evident on a shorter time scale for field data. Thus, immune selection acts to promote persistence of gene combination clusters over epidemiological time, in addition to persistence of the genes themselves over evolutionary time [2, 30].

In ecology, stabilizing competition between species in ecosystems and operational taxonomic units in microbiomes was also shown to promote coexistence and the formation of clusters, albeit for much lower-dimensional trait spaces [7–10]. Here, modules are effectively niches in human immune space, formed by competition for a substitutable resource (naive immune systems). Hence, competition acts to create and fill available niches dynamically [31]. Conceptual similarities and differences in deviations from neutral expectations and in the formation and detection of niches should enrich our understanding of the role of interactions in ecological and epidemiological systems.

Earlier strain theory proposed the stable coexistence of genetically discordant antigenic sets motivated by several pathogens, including malaria, H3N2 influenza, and meningococcus [32–34]. This predicted pattern proved robust in a variety of models depending on the strength of competition (and also the speed of evolution [34]). Unlike earlier computational implementations of strain theory (for example, MANTIS [35]), our ABM is stochastic, incorporates evolutionary innovation, and considers sequential presentation of antigenic variants to the immune system. This formulation was needed to reach diversity comparable to that observed in nature, especially under high endemism. For the large diversity of multigene families such as *var*, neither the complete nor the partial discordance of antigenic repertoires from simpler models is evident.

While for pathogens such as influenza, genetically discordant antigenic sets can represent different strains [33, 34], for malaria, it remains unclear what constitutes a “strain” in regions where antigenic diversity, transmission, and recombination are all high. The genetic coherence apparent in the emergence of persistent niches in antigenic space suggests that the modules we have identified can play the role of strains. As such, strains are dynamic entities arising from the interaction between parasite genomes and the human immune system. Although generated by very different mechanisms, these modules are conceptually similar to viral quasispecies in which high mutation rates and negative selection by host immunity create continuously changing repositories of viral variants, which are the source of virus adaptability [36].

The modular structure we find in the immune selection scenario implies a slower acquisition of immunity by individual hosts (compared to neutrality), demonstrating the epidemiological and evolutionary advantages of immune selection for the parasite. Duration of infection is a key epidemiological parameter, which together with transmission rate, ultimately determines *R*_0_. There is clear empirical evidence for the relationship between PfEMP1 expression and duration of infection. As the major antigen of blood stage, PfEMP1 elicits variant-specific immune responses that increases with age and exposure [37]. In vivo studies [38, 39] show that the whole population of parasites inside a host usually expresses only one or a few dominant *var* genes, with a moderate switching rate per generation. Although the exact genotype–phenotype map of the Duffy binding-like-alpha (DBL*α*) domain analyzed in our empirical data is unknown, this domain is known to help bind to complement receptor (CR)1 and AB blood group antigens on other erythrocytes and form rosetting [40]. Thus, variation of this domain within PfEMP1 is relevant to understanding the chronic nature of malaria infections. The relationship between sequential expression and duration of infection is one mechanism resulting in frequency-dependent selection. We conjecture that regardless of the specific mechanism, a temporal modular structure would emerge when frequency-dependent selection is operating, even in models in which all antigens are seen by the host at once.

Existing epidemiological models of malaria are able to incorporate aspects of repeated infection but neither explicit strain diversity per se nor its overlap structure [41]. How to account for these aspects in transmission models that remain sufficiently parsimonious for epidemiological application remains an open question. Consideration of dynamic strains (or modules) provides one way forward even though the definition of modules may be context-dependent (for example, depend on model parameters). In Senegal, where extensive control efforts have shrunk the parasite population considerably, parasites from the same genetic clone (defined using neutral markers) express similar dominant *var* genes and have similar responses to growth-inhibitory antibodies [42]. These findings appear consistent with our theoretical results, although follow-up work should consider responses to intervention.

Incorporating diversity and its structure may prove particularly relevant to modeling interventions. Control efforts can lower diversity either directly by clearing infections (for example, antimalarial drugs) or through reduction in transmission by targeting the vector (for example, bed nets or IRS). Our simulations and data clearly show that bottlenecks to transmission (and associated generation of *var* diversity), such as seasonality, limit the persistence of modules, while the replenishment and the restructuring of diversity during the following wet season create new ones (S9 Fig). Immune selection can counter the effects of seasonality and/or interventions by promoting persistence of modules across these bottlenecks (S10 Fig). This kind of resilience implies that upon lifting the intervention, the remaining diversity should be able to quickly restructure itself to occupy available niches in immune space. A recent study in Northern Ghana following 7 years of IRS has shown a consistent reduction in EIR and sporozoite rates compared to a nearby control site [43]. Yet, the same study also showed that upon removing the IRS intervention, EIR increased from 30 to 90 infectious bites per person per month in only 2 years. Monitoring *var* gene diversity and its structure may provide further insight, as a reduction in epidemiological parameters per se may not be a sufficient condition for elimination. Previous arguments on multiple fixed strains and *R*_0_, coupled with our findings on structure and infection duration, suggest the existence and raise the open question of a threshold in antigenic (and associated genetic) diversity in the transition to elimination.

One possible limitation of the approach taken here is the assumption of functional equivalence of *var* genes in the model. Previous studies have shown that variation in upstream sequences [40] or homology blocks [17] result in functional differences among *var* genes. This could affect the signature of immune selection. We therefore limited our analyses of empirical data to groups of upstream promoter (ups)B/C to ensure most *var* variants are by and large functionally equivalent. In the future, we will expand our theoretical and computational approach to include variation in virulence or other functions across *var* genes. Another limitation is the potential sensitivity to the community detection method we used. While this can, in principle, be true, the patterns we obtain are sensible from a theoretical perspective. Also, on the technical side, there is currently no other method available to perform community detection in directed and weighted networks with directed interlayer edges in large networks. Because quantitative network properties such as the number of modules can be sensitive to the exact parameterization of the algorithm's implementation or to the cutoff imposed on network edge weights, we emphasize that we are primarily interested in the qualitative differences between the immune selection and neutral scenarios.

From this qualitative perspective, temporal multilayer networks present an opportunity to extend the scope of current phylodynamic theory, which relates the epidemiology and transmission of pathogens to phylogenetic structure [44, 45]. Because trees are a particular case of networks, the multilayer network approach we devise here can extend traditional phylodynamic analysis to pathogens evolving through recombination. As in phylodynamics, the structure of our network reveals information about the interplay between selective forces and transmission dynamics. There is, in particular, a clear difference in structure between the immune selection and neutral scenarios. We also find differences in network structure between regimes with different diversity, whereby low diversity is characterized by a replacement regime instead of module coexistence (S1 Text)—patterns that reflect differences in transmission dynamics and evolutionary rates between these regimes.

Our findings are generally relevant for pathogens characterized by large antigenic spaces. One extreme example is found in *Trypanosoma brucei*, the causal agent of African sleeping sickness. Its genome contains about 1,000 copies of the variant surface glycoprotein gene, the vast majority of which are pseudogenes. This enormous within-genome diversity provides a clear advantage to the parasite, allowing it to assemble new active genes from pseudogenes via gene conversion once it has exhausted its functional copies during an infection [46]. Other examples are found in the fungus *Pneumocystis carinii* and in the bacterium *Neisseria gonorrhoeae* [3]. Beyond multicopy family genes, the theory and analyses we have developed should also apply to sets of genes encoding different antigens.

Competition for hosts is a powerful feature of parasites' life history, enabling the emergence and maintenance of a dynamical strain structure across bottlenecks of transmission and affecting the rate at which host immunity is gained. For malaria, understanding the consequences of parasite population structure for elimination constitutes an important direction for future research.

## Methods

### Ethics statement

The study was reviewed and approved by the ethics committees at the Navrongo Health Research Centre, Ghana (approval #NHRC IRB-131); Noguchi Memorial Institute for Medical Research, Ghana (approval #NMIMR-IRB CPN 089/11-12); University of Melbourne, Australia (approval #HREC 144–1986); and the University of Chicago, United States (approval #IRB 14–1495).

### ABM

The ABM is flexible enough to include seasonality and methods for malaria control to compare simulated dynamics to empirical data (see below). We used an improved implementation of the ABM developed by [15]. A complete description of the model can be found there. Here, we briefly describe the main elements of the model.

#### Structure of human and parasite populations

The human population in the ABM has a fixed size of *H* = 10,000, with a given age structure described by an exponential distribution with a mean life span of 30 years. Hence, whenever a host dies, another is born. The genome of one individual *P*. *falciparum* parasite was considered as a repertoire consisting of a set of 60 not necessarily unique *var* genes, *R* = {*g*_1_,*g*_2_,…,*g*_*l*_} where 1≤*l*≤60 is the index of the gene within the repertoire and *g*∈*U*[1,*G*] is the ID of the gene in a pool of *G* genes. Each *var* gene was composed of two loci, corresponding to two epitopes. We refer to the variants of each locus as alleles hereafter. This model of *var* genes is supported by empirical evidence for two hypervariable regions bounded by three semiconserved regions [47, 48]. Hence, each gene is defined as $g=\{a_{1}^{k},a_{2}^{m}\}$, where the superscripts *k*,*m*∈*U*[1,*A*] are the ID of the allele in a pool of *A* alleles (the two epitopes have separate pools that are equal in size) and the subscript defines the epitope. A focal population of the parasite is initialized from an external gene pool representing regional diversity. New genomes with randomly selected genes from the regional gene pool enter the system with an immigration rate of 1 genome per day, and a random host is selected and infected with the new genome. Each simulation starts with 20 infected hosts, each with one repertoire selected at random from the pool.

#### Transmission dynamics and seasonality

We set a biting rate *b* so that the average waiting time to the next biting event is equal to 1/(*b* × *H*). When a biting event occurs, two hosts are randomly selected, one donor and one recipient. If the donor has infectious parasite repertoires, the receiver will be infected with a probability *p* (the transmission probability). If the donor is infected with multiple repertoires in the blood stage, then the transmission probability of each strain is reduced to *p*/*I*, where *I* is the number of repertoires.

We implemented seasonality by multiplying *b* by a temporal vector *v* of length 360 (days), containing the daily number of mosquitoes, resulting in a temporal vector of daily biting rates *ν* = ***v***×*b*. To obtain ***v***, we used the deterministic model for mosquito population dynamics from [49]. The model was originally developed for *Anopheles gambiae* and consists of a set of ODEs describing the dynamics of 4 mosquito stages: eggs, larvae, pupae, and adults. Seasonality is implemented via density dependence at the egg and larva stages as a function of rainfall (availability of breeding sites). The model can also handle IRS interventions for comparison to empirical data. We emphasize that the values in the biting rate vector *v* rather than the absolute number of daily mosquitoes are the key parameter for the purposes of this work, used to match the EIR observed in the study area to that of the simulations. Because implementing seasonality requires some knowledge on the dynamics of mosquitoes and on the resulting EIR, we only implemented seasonality for the high-diversity regime. Details on the model can be found in [49], and our implementation (in Mathematica) at the GitHub repository.

#### Mechanisms of genetic change

During the sexual stage of the parasite (within mosquitoes), different parasites can exchange *var* repertoires through meiotic recombination. The receiver host can receive either recombinant repertoires, original repertoires, or a combination. During the asexual stage of the parasite (blood stage of infection), *var* genes within the same repertoire can exchange epitope alleles through mitotic (ectopic) recombination (at a rate of *ρ* = 1.87×10^−7^ per gene pair per day [23]). Also, new alleles of each epitope can be generated through mutations at a rate of *μ* = 1.42×10^−8^ per day. Mitotic recombination, mutation, and immigration generate new *var* genes [23].

#### Within-host dynamics

Each repertoire is individually tracked through its entire life cycle, encompassing the liver stage, asexual blood stage, and the transmission and sexual stages explicitly or implicitly. Because we do not explicitly model transmission via mosquitoes, we delay the expression of each strain in the receiver host by 14 days to account for the time required for the sexual stage (in the mosquito) and the liver stage (in the host). Specifically, the infection of the host is delayed 7 days to account for the time required for gametocytes to develop into sporozoites in mosquitoes. When a host is infected, the parasite remains in the liver stage for an additional time before being released as merozoites into the bloodstream, invading red blood cells, and starting the expression of the *var* repertoire.

During the expression of the repertoire, the host is considered infectious with the active repertoire. The expression order of the repertoires is randomized for each infection, while the deactivation rates of the *var* genes is controlled by the host immunity. When one gene is actively expressed, host immunity “checks” whether it has seen any of its epitope alleles in the infection history. In the immune selection model, the deactivation rate changes so that the duration of active period of a gene is proportional to the number of unseen alleles. A gene deactivates at a rate of 1/6 per day in a naive host. After the gene is deactivated, the host adds its deactivated alleles to the immunity memory. A new gene from the repertoire then becomes immediately active. The repertoire is cleared from the host when the whole repertoire of *var* genes is depleted. The immunity towards a certain epitope allele wanes at a rate *w* = 1/1,000 per day [50].

### Scenarios

In the immune selection scenario, the duration of infection in a host depends on the history of infection with given *var* genes (or their alleles) as described above. The model of “complete neutrality” retains the process of transmission but does not consider any aspect of infection history. Hence, individuals clear infection after a given amount of time (matched to the average duration of infection from counterpart simulations of the selection scenario). In the neutral model of “generalized immunity,” the duration of infection depends on the number of previous infections but not on their specific genetic composition. For a meaningful comparison, we parameterized this model to match the curve for duration of infection with previous number of infections of the immune selection scenario [15].

In both neutral models, we ran the exact same ABM implementation of the immune selection scenario, conserving all its parameters except for the duration of infection. Specifically, we used *G* = 12,000 and *b* = 0.5 in all simulations in the high-diversity regime (medium diversity: *G* = 1,200, *b* = 0.2; low diversity: *G* = 120, *b* = 0.1). The duration of infection of a repertoire in a naive host was D = 360 days (i.e., 1 year). Because we know that in nature, hosts build specific immunity to malaria infections, the neutral models can be regarded as process-based null models against which to test the structure obtained with the full model (with immune selection) and that of the empirical data. These null models lack immune selection/competition for hosts via specific immunity and have either no competition in the complete neutrality formulation or general competition for the overall host population in the generalized immunity one. For each combination of diversity regime (low, medium, high) and immune scenario (immune selection, complete neutrality, and generalized immunity), we analyzed data generated by 50 simulations of the ABM.

### Construction of temporal networks

We calculated genetic similarity of repertoire *i* to repertoire *j* as *S*_*ij*_ = (*N*_*i*_∩*N*_*j*_)/*N*_*i*_, where *N*_*i*_ and *N*_*j*_ are the number of unique alleles for repertoires *i* and *j*, respectively (the genetic similarity of repertoire *j* to repertoire *i* was calculated as *S*_*ji*_ = (*N*_*i*_∩*N*_*j*_)/*N*_*j*_. We used a directional index because of the asymmetric competition resulting from different numbers of unique alleles in a repertoire [15]. We used the same index (*S*) for both intra- and interlayers because it situates the inter- and intralayer edges on the same scale, which is crucial when looking for optimal partitioning in multilayer networks [51, 52]. To optimize the signal-to-noise ratio in our analysis, we imposed a cutoff on edge weights (S1 Text and S3 Fig).

### Community detection

To capture the organization of the population into groups of highly similar repertoires, we used the map equation as an objective function to calculate the optimal partition of the network. Briefly, the map equation is a flow-based and information-theoretic method (implemented with Infomap) recently extended to multilayer networks that calculates network partitioning based on the movement of a random walker on the network (see [26–28] for details). In any given partition of the network, the random walker moves across nodes in proportion to the direction and weight of the edges. Hence, it will tend to stay longer in dense areas where there are many repertoires similar to each other. These areas can be defined as “modules.” Note that for convenience, we use the term “modules” because it is commonly used to refer to partitions of networks, but Infomap does not calculate a modularity function (sensu [53]). The time spent in each module can be converted to an information-theoretic currency using an objective function called the map equation. The best network partition corresponds to that with the minimum value of the map equation [26, 27]. This method has been applied to describe temporal flows in networks that do not have interlayer edges [54]. In our particular network, once the random walker moves along an interlayer edge, it cannot go back, capturing the temporal flow in the network.

### Quantification of structure

We calculated the relative persistence of repertoires and modules as $P=(l_{d}-l_{b})/(l_{max}-l_{b})$, where *l*_*b*_ and *l*_*d*_ refer to the layers in which a module (or repertoire) first appeared or died, respectively and *l*_*max*_ is the maximum number of layers (*l*_*max*_ = 300 in our simulations). This index has a maximum theoretical value of 1 when a module (repertoire) persists from the layer when it was generated till *l*_*max*_ and a theoretical minimum of 1/(*l*_*max*_−*l*_*b*_) when a module (repertoire) persists for a single layer. We used the relative persistence instead of the plain number of layers in which a module (repertoire) persisted because persistence can be truncated artificially by reaching the end of the simulation. For example, a repertoire could have persisted for 60 layers because it appeared in layer 100 and died in layer 160 (P=0.3) or because it appeared in layer 240 and died only because it reached the end of the simulation at *l*_*max*_ = 300 (P=1). In the former case, the repertoire did not live to its full potential (200 layers) and thus has lower relative persistence than the second one. We also examined the results for absolute persistence (i.e., the number of layers), and these do not qualitatively change the conclusions.

We calculated *J*, the evenness in module size in each layer. We chose evenness instead of simply considering the number of repertoires in a module because the number of repertoires is not comparable between scenarios and because from a theoretical perspective, limiting similarity resulting from selection should promote an even distribution of repertoires across modules. We calculated *J* as *J* = *H*′/*H*_*max*_ [55]. $H^{\prime }=-\sum_{i=1}^{m}p_{i}ln(p_{i})$ is Shannon's diversity, where *m* is the total number of modules in the layer and *p*_*i*_ is the proportion of repertoires in module *i* in that layer. *H*_*max*_ = *ln*(*m*) is the maximum diversity possible, obtained when all modules have the same size [55]. *J* varies between 0 and 1, where 1 indicates a perfect distribution of module sizes such that each repertoire is as different from any other as possible.

### Empirical data

The empirical data were collected from a study of two catchment areas in BD, Ghana, located in the Upper East Region near the Burkina Faso border. This age-stratified serial cross-sectional study was done across 6 Svs (Sv1–Sv6), conducted at either the EWS or EDS: October 2012 (EWS, Sv1), May–June 2013 (EDS, Sv2), May–June 2014 (EDS, Sv3), October 2014 (EWS, Sv4), October 2015 (EWS, Sv5), and May–June 2016 (EDS, Sv6). Each survey lasted approximately 3–4 weeks. Details on surveys are in S1 Table. During the sampling period, three rounds of IRS intervention were performed: between October–December 2013, between May–July 2014, and between December 2014 and Feb 2015, using organophosphates. We sequenced and genotyped part of the *var* DBL*α* domain, which is a reliable marker for differentiating *var* genes.

*Var* genes can be divided into subgroups based on their chromosomal location and semiconserved ups sequences: groups A (upsA), B (upsB), C (upsC), and E (ups E). [56]. Different ups *var* gene groups are associated with differences in disease virulence and sequence conservation; in particular, upsA *var* genes are more conserved (i.e., have lower genetic variation at the population level) and are preferentially expressed in children with severe and/or clinical malaria compared to upsB/upsC *var* genes. Hence, DBL*α* reads were clustered at 96% pairwise identity, translated into all 6 reading frames, and classified into either upsA or upsB/upsC (i.e., non-upsA) groups [19]. Details on the study population, data collection procedures, molecular and genetic work, and epidemiology have been published elsewhere [15, 29]. While the *var* gene data from Sv1 and Sv2 were used in [15], data from Sv3–Sv6 are used here for the first time and are summarized in S1 Table.

### Comparison of empirical and simulated benchmark networks

The DBL*α* tag region consists of two hypervariable regions and one conserved region between the S2 and S3 domains [48]. Therefore, we split each tag sequence before and after the HB5 block into two hypervariable regions using USEARCH blast and considered them as two epitopes. We clustered each epitope sequence into different alleles using 80% similarity to match the mean number of alleles in a layer in the ABM simulations. As in our simulations, we constructed each layer in the empirical network based on pairwise similarities of repertoires consisting of different alleles of the two epitopes. Only upsB/upsC DBL*α* tags were included because upsA genes tend to be more conserved and thus have disproportionately higher sharing rates among repertoires [19]. Since infections with multiple parasite genomes (multiplicity of infection [MOI] > 1) are very common in malaria-endemic regions, we selected isolates with a total number of upsB/upsC DBL*α* types ranging from 40–55 copies to maximize the probability of selecting hosts with single-genome infections (MOI = 1). This resulted in 90, 68, 69, 52, 115, and 44 isolates in layers 1–6, respectively. We defined edge weights using the same index as in the theoretical work and used a cutoff of 97.8% (S11 Fig and S12 Fig).

We note, based on our preliminary analyses, that constructing an empirical network is not as straightforward as for the benchmark theoretical networks because of the uneven sampling interval between the surveys. One way to account for uneven sampling is to rescale the interlayer edges. Any rescaling entails, however, assumptions about processes underlying repertoire persistence. One approach based on genetic drift would require knowledge on the population demography of the parasite, which we do not have. Another would rely on the time scale of the processes influencing similarities between repertoires that include the consecutive sampling of the same repertoires across time, the generation of a new copy of a repertoire, and the similarity between recombinant repertoires. The relative importance of these processes is difficult to estimate. On the more technical side, rescaling may place the intra- and interlayer edges on different scales, which could impose different weights in how the clustering algorithm (Infomap) treats the relative importance of similarity between and within layers [52]. Finally, rescaling is applied only to interlayer edges, giving them a different interpretation than that of intralayer edges. Given these limitations, we opted for not rescaling the interlayer edges, especially because the relatively long persistence of epitope alleles (66% persist for 3 or more layers) reduces the need to account for uneven sampling periods, making assumption-dependent corrections unnecessary.

We ensured that our results of the theoretical part were qualitatively the same when seasonality was considered. To imitate the empirical setting, we ran 50 ABM simulations with *G* = 35,000 and a vector of biting rates *v* that generated EIR values similar to those observed in the field (S2 Fig and S8 Fig). Because of the uncertainty in realistic parameter values of the ABM for the given field sites, we based our comparisons to empirical data on 100 simulations that span plausible variation across the two main parameters:

1. Average biting rate, *b*. We tested 10 sequential values of *b*: 0.00010, 0.00012, 0.00014, 0.00017, 0.00019, 0.00021, 0.00023, 0.00026, 0.00028, and 0.00030. In our simulations in the main text, *b* = 0.00002. Remember that effective biting rate is obtained by *ν* = ***v***×*b* (see description of the ABM above); we kept ***v*** constant.
2. Size of the *var* gene pool, *G*. We tested 10 sequential values of *G*: 30,000, 31,111, 32,222, 33,333, 34,444, 35,556, 36,667, 37,778, 38,889 and 40,000. In our simulations in the main text, *G* = 35,000.

Beyond seasonality, we included the IRS by matching the relative differences in biting rates to those obtained in the field. We accounted for the three rounds of IRS by reducing the biting rates in the mosquito model (see description of the ABM above) to obtain EIR values of approximately 0, as was recorded in the field, without collapsing the mosquito population. In each of the 100 ABM runs, we selected 6 layers corresponding to the 6 months in which surveys were conducted in the field (encompassing the interventions). We then subsampled these layers randomly to obtain the same number of repertoires as in each layer of the empirical network. We used *var* epitopes as the unit of comparison to define edge weights, as was done for the empirical networks and the theoretical work. In these 100 networks, we imposed the same 97.8% cutoff that was used in the empirical network (S11 Fig). Given the sensitivity of the analysis to the particular way one builds the empirical network (and consequently, the counterpart theoretical networks), one may fail to find differences in the temporal modular structure between generalized immunity and immune selection given short and uneven longitudinal sampling.

For any module that was “born” in layer 3 or before, we calculated the probability that it persisted for at least 3 layers using a logistic regression implemented in the function glm with a binomial family in R. Because our explanatory variable is categorical with 4 levels (empirical data and three scenarios), the model has a set of “dummy variables” and can be written as *ln*(*y*) = *β*_0_+*β*_1_*x*_1_+*β*_2_*x*_2_+*β*_3_*x*_3_+*ε*, where *y* is 1 if the module persisted or 0 otherwise, *β*_0_ is the coefficient for the empirical data, and *β*_1_,*β*_2_,*β*_3_ are the coefficients for immune selection, generalized immunity, and complete neutrality, respectively.

### Simulations of host infection

We simulated repeated infections of naive hosts by sampling repertoires originating in the same module, in different modules, or regardless of modules. We compared the resulting curves depicting the decrease in the duration of infection as a function of the number of previous infections for each of these dynamics. In these simulations, infection takes place within a layer for a given number of bites determined by a given force of infection and then moves on to the next layer. In this way, we explicitly consider different times. Details on the simulations are in S1 Text.

## Supporting information

S1 Text Additional details on methods and results. The text includes methodological details on selection of cutoff values and the epidemiological simulations, results for low- and medium-diversity regimes, and a sensitivity analysis.
(PDF)

S1 Fig Characterization of diversity regimes. The left, middle, and right columns represent the low-, medium-, and high-diversity regimes, respectively. Each row depicts one parameter relevant for epidemiology or genetic diversity. Whereas parameters that are set a priori in the model (number of bites, number of alleles) have similar values between scenarios, emerging parameters (for example, the number of repertories, prevalence, or MOI) can differ. These regimes apply to the simulations in the theoretical analyses. Data are for 50 ABM simulations. Data from model output used to produce the figure can be found in https://figshare.com/articles/Figure_data/8079713. ABM, agent-based model; G, generalized immunity; MOI, multiplicity of infection; N, complete neutrality; S, selection.
(PDF)

S2 Fig EIR in ABM simulations. Panels (A) and (B) show the EIR for simulations without and with seasonality, respectively, for the 3 scenarios: immune selection (red), generalized immunity (blue), and complete neutrality (orange). In (A), each panel depicts a different transmission regime. Because there is no seasonality, EIR does not vary across months. In (B), values are for simulations with seasonality in the high transmission regime only, and each panel depicts a different scenario. Data from model output used to produce the figure can be found in https://figshare.com/articles/Figure_data/8079713. ABM, agent-based model; EIR, entomological inoculation rate.
(PDF)

S3 Fig Determination of cutoff values for networks. The left, middle, and right columns correspond to the low (teal), medium (purple), and high (green) diversity regimes, respectively, in the immune selection scenario. (A–I) Change in values of three network properties as a function of cutoff percentile (see Methods for details on how we calculated these properties). In each cutoff value, we draw a box plot and mean (red point) for values across 10 simulations of the ABM. We selected cutoff percentiles of 0.3, 0.6, and 0.85 in the low, medium, and high regimes, respectively. The same percentiles were used in the counterpart neutral and generalized immunity model scenarios. Data from model output used to produce the figure can be found in https://figshare.com/articles/Figure_data/8079713. ABM, agent-based model.
(PDF)

S4 Fig Temporal population structure in the low-diversity regime. The left, middle, and right columns represent the selection (red), generalized immunity (blue), and neutral (orange) scenarios, respectively. (A,B,C) Example of population structure from one run of the ABM. Each line on the *y*-axis corresponds to a different module, and its horizontal length depicts its occurrence across layers. Modules are generated and die out. All scenarios are characterized by replacement dynamics whereby one module persists until it is replaced by another, possibly following a short transition period. (D,E,F) These dynamics result in module persistence (colored density plots), which is shorter than that of repertoires (gray density plots) in all scenarios. The insets depict a comparison between the values in the colored and gray density plots using box plots. See Methods for details on how we calculated relative persistence. Results in (D)–(E) are for data pooled across 50 runs of the ABM. Data from model output used to produce the figure can be found in https://figshare.com/articles/Figure_data/8079713. ABM, agent-based model.
(PDF)

S5 Fig Temporal population structure in the medium-diversity regime. The left, middle, and right columns represent the selection (red), generalized immunity (blue), and neutral (orange) scenarios, respectively. (A,B,C) Example of population structure from one run of the ABM. Each line on the *y*-axis corresponds to a different module, and its horizontal length depicts its occurrence across layers. Modules are generated and die out. Under complete neutrality, the persistence of repertoires is on the same scale as that of modules, as expected (F). However, there is no apparent difference in population structure and patterns of relative persistence between immune selection and generalized immunity (D,E) (see results for low- and medium-diversity regimes in S1 Text for details). In panels (D)–(F), the insets depict a comparison between the values in the colored and gray density plots using box plots. See Methods for details on how we calculated relative persistence. Results in (D)–(E) are for data pooled across 50 runs of the ABM. Data from model output used to produce the figure can be found in https://figshare.com/articles/Figure_data/8079713. ABM, agent-based model.
(PDF)

S6 Fig Evenness in module size. Limiting similarity resulting from selection should promote an even distribution of repertoires across modules. We calculated the evenness *J* in each layer as *J* = *H*′/*H*_*max*_ [55], where *H*′ is Shannon's diversity and *H*_*max*_ is the maximum diversity possible, obtained when all modules have the same size [55]. *J* varies between 0 and 1, where 1 indicates a perfect distribution of module sizes such that repertoires are at the “limit” of their limiting similarity because each repertoire is as different from any other as possible. We compare between scenarios using box plots for the low- (A), medium- (B), and high-diversity regimes (C). Because immune selection does not operate in the low-diversity regime, the evenness is comparable across scenarios. By contrast to high diversity, competition under medium diversity is not strong enough to generate patterns that distinguish between immune selection and generalized immunity. (D) For high diversity, evenness is higher when there are more modules in a layer. Results are for data pooled across 50 runs of the ABM. Data from model output used to produce the figure can be found in https://figshare.com/articles/Figure_data/8079713. ABM, agent-based model; G, generalized immunity; N, complete neutrality; S, Immune selection.
(PDF)

S7 Fig Sensitivity analysis for high-diversity regime. We repeat the analysis of the theoretical part of the main text using 112 simulations for the immune selection (red) and generalized immunity (blue) scenarios. These 112 simulations encompass variation in three key parameters of the ABM. We find the same qualitative results for the differences between the two scenarios as in the main text. Specifically, the relative persistence of modules (𝒫) is longer (A), and the evenness in module size is higher (B) under immune selection compared to generalized immunity. Data from model output used to produce the figure can be found in https://figshare.com/articles/Figure_data/8079713. ABM, agent-based model.
(PDF)

S8 Fig Characterization of the seasonality diversity regime. The left, middle, and right columns represent the selection, generalized immunity, and complete neutrality scenarios, respectively. Each row depicts one parameter relevant for epidemiology or genetic diversity. This regime is for the baseline simulations of seasonality, with *G* = 35,000, used to confirm that our nonseasonal theoretical results hold. Data are for 50 ABM simulations. Data from model output used to produce the figure can be found in https://figshare.com/articles/Figure_data/8079713. ABM, agent-based model.
(PDF)

S9 Fig Seasonal signature in the formation of modules. Seasonality promotes the extinction of repertoires in the dry season and the generation of new ones in the wet season. This bottleneck causes a decrease in the creation of new niches, translating to a decrease in the number of modules in the dry season. Data are pooled across 50 runs of the ABM. Error bars represent 95% confidence intervals. Red: immune selection; blue: generalized immunity; orange: complete neutrality. Data from model output used to produce the figure can be found in https://figshare.com/articles/Figure_data/8079713. ABM, agent-based model.
(PDF)

S10 Fig Temporal population structure in the high-diversity regime with seasonality. The left, middle, and right columns represent the selection (red), generalized immunity (blue), and neutral (orange) scenarios, respectively. (A,B,C) Example of representative population structure from one run of the ABM. Each line on the *y*-axis corresponds to a different module, and its horizontal length depicts its occurrence across layers. Modules are generated and die out. (D) The selection scenario is characterized by modules (red) that persist for much longer than the repertoires (gray) that compose them. The inset is a comparison between the values in the red and gray density plots using box plots. (E,F) In the neutral scenarios, modules and repertoires have similar relative persistence. See Methods for details on how we implemented seasonality and calculated relative persistence. Results in (D)–(E) are for data pooled across 50 runs of the ABM. Data from model output used to produce the figure can be found in https://figshare.com/articles/Figure_data/8079713. ABM, agent-based model.
(PDF)

S11 Fig Exploration of cutoff for empirical networks. Because empirical networks are not necessarily identical to those produced in our theoretical work (for example, in size and genetic diversity), we explored the effect of thresholding. It is clear from the figure that cutoff does not have a major effect on the distribution of module persistence. We chose 97.8% because this was the point of transition at which modules started to persist for 5 layers. Data used to produce the figure can be found in https://figshare.com/articles/Figure_data/8079713.
(PDF)

S12 Fig Comparison of edge weight distributions between empirical and simulated networks. We assess how well our simulations for the immune selection scenario (red) matched the empirical data (purple) by comparing the distributions of edge weights. The lower and upper panels depict the distributions for the intra- and interlayer edges, respectively. Vertical lines represent the values where a cutoff of 97.8% was applied. Data from model output used to produce the figure can be found in https://figshare.com/articles/Figure_data/8079713.
(PDF)

S13 FigEpidemiological model curve fits.The lines depict the predicted values for duration of infection as a function of host age. The values were obtained for the exponential model $d=ae^{-b_{i}}$ (where *d* is the duration of infection and *i* is the number of previous infections) that was fitted to the data from Fig 4D in the Main text. We used the EIR of each scenario and the number of accumulated infections to calculate host age. Curves of the standard error overlap with the curves of predicted values and are not presented (the standard error of model coefficient is very small). Red: Immune selection; blue: generalized immunity; orange: complete neutrality. Data used to produce the figure can be found in https://figshare.com/articles/Figure_data/8079713. EIR, entomological inoculation rate.(PDF)

S1 Table Summary of empirical data relevant for this study. Extended details are in [15, 29]. Isolates are the number of people in which the parasite was detected by microscopy. Unique *var* types is the total number of genes obtained from the DBL*α* marker with the standard 96% similarity cutoff. Alleles is the number of alleles from the UpsBC *var* gene group that were detected in isolates with a single infection (MOI1; defined as isolates from which 40–55 *var* UpsBC genes were sampled). The last two columns describe the data used in the empirical analysis in the main text, with MOI1 being the number of nodes. DBL*α*, Duffy binding-like-alpha; MOI, multiplicity of infection; ups, upstream promoter.
(PDF)

## Abbreviations

ABM: agent-based model
BD: Bongo District
CR1: complement receptor 1
DBL*α*: Duffy binding-like-alpha
EDS: end of dry season
EIR: entomological inoculation rate
EWS: end of wet season
IRS: indoor residual spraying
MOI: multiplicity of infection
PfEMP1: *P*. *falciparum* erythrocyte membrane protein 1
Sv: survey
ups: upstream promoter
