# Supplementary material for: Competition for hosts modulates vast antigenic diversity to generate persistent strain structure in Plasmodium falciparum
Source: PLoS Biol. 2019 Jun 24;17(6):e3000336. doi: 10.1371/journal.pbio.3000336 (PMC6611651; doi:10.1371/journal.pbio.3000336)
Supplement: S1 Text — The text includes methodological details on selection of cutoff values and the epidemiological simulations, results for low- and medium-diversity regimes, and a sensitivity analysis. (PDF) [file pbio.3000336.s001.pdf]

Text S1

This document contains additional methods, results and all the supporting figures.

Cut-off of edge weights

Two repertoires that share at least one epitope will be connected in the network. This can make networks highly dense as the result of many weak links that indicate weak competition. One challenge inherent to the analysis of networks of this kind is to find the cutoff in edge values that provides the best ratio of signal to noise when studying their structure. On the one hand, too low a cutoff will retain edges that do not significantly affect interactions (here, competition); on the other hand, too high a cutoff will over-fragment the network. To capture the signal most meaningful to reveal interactions, we calculated (i) module's relative persistence, $\mathcal{P}$, (ii) the number of modules per layer (across 300 layers); and (iii) the evenness in the size of modules in a given layer, J (see Methods in Main Text), across a range of cutoff percentiles ([0.25,0.95] in increments of 0.05), separately for each regime for 10 runs of the agent-based model ABM (S3 Fig). We used 10 runs because the sweep across cutoffs is computationally intensive and because the error (around the mean) we obtained in this analysis was rather low even for 10 simulations. We selected the cutoff that best represented a discontinuity or transition (if present) in the values of these variables. This value was obvious in high diversity but less so in low and medium diversity. Therefore, we retained the edges with weights equal or above the 0.3, 0.6 and 0.85 percentile in the low, medium and high regimes, respectively.

Simulations of host infection

We tested the epidemiological consequences of module structure using a simulation experiment to address the acquisition of immunity and its effect on the duration of infection. We specifically simulated repeated infections of naïve hosts with repertoires originating in the same module (*within-module* infections), in different modules (*between-module* infections) or regardless of module (*random* infections). We compared between curves depicting the decrease in the duration of infection as a function of the number of previous infections in each of these conditions. This procedure assumes the same rate of arrival of infectious bites in populations structured by different processes (selection, generalized immunity and complete neutrality). Note that this epidemiological simulation is not performed within the ABM but rather samples from the networks resulting from the ABM (50 networks of high diversity regime).

Infection dynamics. The infection takes place within a time layer for a given number of bites, L, and then moves on to the next layer, explicitly incorporating the temporal component of the system. We used EIR, defined as the number of infectious bites a host receives per day, to inform the algorithm on the number of infections per layer. EIR is a quantity that emerges as a function of transmission dynamics and genetic diversity, and is not set a priori. It is defined as $E = fb$, where f is the proportion of infectious bites (obtained for each run of the ABM) and b is the value of the input parameter defining biting rate in the ABM. Because a layer represents a snapshot of infections accumulated during 30 days, there are $L = 30 \cdot E$ infections in every layer. For instance, if $E = 0.5$, there would be $L = 30 \cdot 0.5 = 15$ infectious bites in each layer (we rounded up fractions). The average EIR values (across 50 ABM simulations) emerging from the simulations were 3, 2.8, and 14 infectious bites per person per month in the selection, generalized immunity and neutral scenarios, respectively. In the within-module dynamics we sampled repertoires within the same module in different layers. In the between-module dynamics we used the same procedure with one difference: Instead of selecting L repertoires from the same module, in any given layer we selected uniformly at random L repertoires from L different modules. In the random infections dynamics we sampled L repertoires per layer uniformly at random regardless of their module affiliation. Because infections were sampled in discrete time with a monthly interval, each layer contains genomes with some accumulation of genetic changes compared to the previous one. Therefore, each time the simulation advances a layer, some repertoires may be new to the host, causing an intermittent increase in the duration of infection. The differences between a previous and current layer are stronger when sampling within-module (and randomly), compared to between-module, because for the latter infections are already largely different by definition.

Calculating duration of infection. In our ABM, the duration of infection of a completely naïve host (e.g., a newborn) was 360 days. Therefore, we defined the duration of infection of each allele of two *var* gene epitopes that the host has not seen before to be $360/60/2 = 3$ days; that is, deactivation of a gene is a Poisson event with a rate of 1/3. This assumes that the duration of infection of each infection is determined solely by the number of alleles it contains to which the host has not been exposed. Although the within-host dynamics of *P. falciparum* is still an open area of study [1], this calculation gives us a general view of the relationship between structure and epidemiology. It allows us to compare immune selection to the two neutral models because the assumption on duration of infection/within-host dynamics is the same for those three scenarios. We infected a host sequentially (in the order repertoires were sampled), following the accumulation of new alleles the host was exposed to with every infection. We calculated the duration of infection for every new infection.

Experimental design. In each temporal network we selected 10 modules that persisted for at least 1 year (12 layers). If there are not enough modules with that persistence (neutral scenarios tend to have modules with short persistence), we restricted our analysis to the ones that did persist. In each of these modules we selected uniformly at random 10 initial layers t to start the repeated infections' simulation. The process of infection then lasts from layer t to $t + 12$ with L infections in each layer and is repeated 10 times to simulate infection in 10 hosts. We simulated host infections in networks resulting from 20 ABM runs (in each of the three scenarios). This resulted in 20,000 infection iterations for each of the within-module, between-module and random infections scenarios.

Results for low and medium diversity regimes

To understand the effect of competition on repertoire population structure we consider regimes of 'low', 'medium' and 'high' diversity/transmission, where the number of *var* genes in a local population is on the order of magnitude of ≈100 (e.g., Latin America), ≈1,000 (e.g., Asia/Pacific) and ≈10,000 (e.g., sub-Saharan Africa), respectively [2–4]. See S1 Fig and S2 Fig for characteristics of the different regimes. In this section we present results for the low and medium diversity regimes.

Low diversity regime. In the low-diversity regime, repertoires were typically grouped into a single module, with brief episodes of reorganization in repertoire composition expressed as module switching (S4A Fig). Low diversity leads to high similarity between repertoires. Hence, immunity to circulating *var* genes accumulates fast in the small infected human population resulting in a low number of susceptible individuals. In low diversity there are fewer opportunities for innovation at the *var* gene and repertoire levels. Hence, the number of *var* genes circulating in the population does not change and balancing selection does not operate because there is not enough diversity to maintain polymorphism. Immune selection therefore removes new repertoires due to cross-immunity, creating sharp boundaries of similarity between existing and newly formed modules. The replacement of modules reflects therefore the reorganization of the circulating *var* diversity rather than the establishment of a new set of *var* genes.

We observed similar replacement dynamics in the neutral scenarios (S4B Fig and S4C Fig) but the mechanisms leading to these dynamics are different. There is no accumulation of immunity in the host population. Hence, the replacement of modules is solely the result of the accumulation of new repertoires, which at some point drift to be sufficiently different to create a new module. The replacement dynamics in the selection and the two neutral scenarios results in modules having shorter persistence than the repertoires (S4D-F Fig).

The lack of selection also results in a similar evenness in all scenarios (S6A Fig).

Medium diversity regime. Increased genetic diversity relaxes the constraints on the number of ways *var* genes can combine to form repertoires, enhancing the number of possibilities to partition the population. This crucial difference in the transition from low diversity to medium/high diversity allows immune selection mediated by human immunity to operate. It creates a qualitative shift whereby the dynamics in medium and high diversity are of module co-existence rather than replacement in the selection scenario. This difference can be clearly seen in a comparison to complete neutrality (S5A and S5C Figs). Nonetheless, there is no apparent difference in population structure between immune selection and generalized immunity (S5A and S5B Figs).

By construction to generate a meaningful neutral model, we match the duration of infection of generalized immunity to that of immune selection. As with any over-constrained null hypothesis, this match may result in a similar structure between the two scenarios. We have already shown, however, that for non-temporal networks it is possible to distinguish between these scenarios based on a suite of network properties other than just modularity [4]. In the temporal setting studied here, we observe a similar population structure from the perspective of modularity in the two scenarios (S5 and S6 Figs). This similar structure results from the fact that the duration of infection is a decaying function, allowing for the existence of some repertoires with high duration. Under generalized immunity, these imposed long-persisting repertoires are able to form modules due to clonal transmission, resembling the non-overlapping niches maintained by competition between repertoires under immune selection. It is highly likely that with a thorough network analysis that considers properties other than modularity we would be able to distinguish between these two temporal networks, given that the mechanisms behind the modules are completely different and membership in a module involves negative selection and not just clonal expansion in the full model. (This distinction would follow from the previously demonstrated differences in much shorter periods [4] but is beyond the scope of this work focused on modularity). Under high diversity, we do not observe similar modular structure between the scenarios because meiotic recombination rates are much higher than in medium diversity (more hosts contain multiple infections), reducing the likelihood that modules would persist simply by clonal transmission under generalized immunity.

Sensitivity of model in high diversity

We compared the sensitivity of the results of our theoretical simulations at high diversity to three parameters:

1. Biting rate, b. We tested 4 sequential values of b: 0.3, 0.4, 0.5, 0.6. In our simulations in the main text, $b = 0.5$.

2. Size of the *var* gene pool, G. We tested 7 sequential values of G: 10000, 11000, 12000, 13000, 14000, 15000, 16000. In our simulations in the main text, $G = 12000$.

3. Duration of infection in a naive host, D. We tested 4 sequential values of D: 180, 300, 420, 540. In our simulations in the main text, $D = 360$ days.

We ran one simulation per combination of these parameters for the selection and generalized immunity scenarios, which are the ones that are potentially difficult to distinguish. We then compared the relative persistence ($\mathcal{P}$) and the evenness (J) of these two scenarios. We find that these scenarios are qualitatively different, with the same trends we find in the main text (S7 Fig).

References

[1] Bruce MC, Donnelly Ca, Alpers MP, Galinski MR, Barnwell JW, Walliker D, et al. Cross-species interactions between malaria parasites in humans. Science. 2000;287:845–848. doi:10.1126/science.287.5454.845.

[2] Barry AE, Leliwa-Sytek A, Tavul L, Imrie H, Migot-Nabias F, Brown SM, et al. Population genomics of the immune evasion (*var*) genes of Plasmodium falciparum. PLoS Pathog. 2007;3(3):e34. doi:10.1371/journal.ppat.0030034.

[3] Day KP, Artzy-Randrup Y, Tiedje KE, Rougeron V, Chen DS, Rask TS, et al. Evidence of strain structure in Plasmodium falciparum var gene repertoires in children from Gabon, West Africa. Proceedings of the National Academy of Sciences. 2017; p. 201613018. doi:10.1073/pnas.1613018114.

[4] He Q, Pilosof S, Tiedje KE, Ruybal-Pesántez S, Artzy-Randrup Y, Baskerville EB, et al. Networks of genetic similarity reveal non-neutral processes shape strain structure in Plasmodium falciparum. Nat Commun. 2018;9(1):1817. doi:10.1038/s41467-018-04219-3.
