# Supplementary figures and images for: Competition for hosts modulates vast antigenic diversity to generate persistent strain structure in Plasmodium falciparum
Source: PLoS Biol. 2019 Jun 24;17(6):e3000336. doi: 10.1371/journal.pbio.3000336 (PMC6611651; doi:10.1371/journal.pbio.3000336)

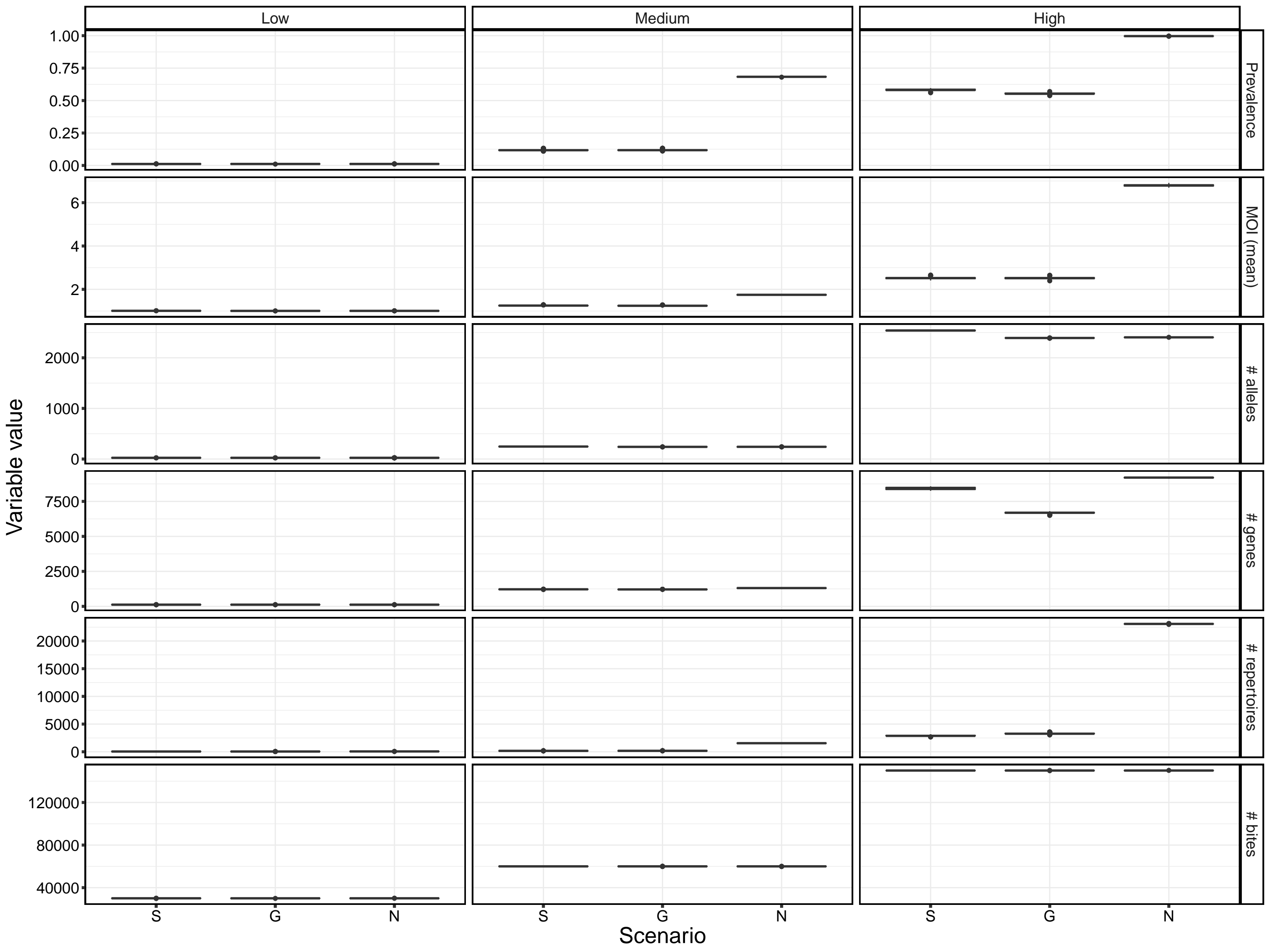

Supplement: S1 Fig — The left, middle, and right columns represent the low-, medium-, and high-diversity regimes, respectively. Each row depicts one parameter relevant for epidemiology or genetic diversity. Whereas parameters that are set a priori in the model (number of bites, number of alleles) have similar values between scenarios, emerging parameters (for example, the number of repertories, prevalence, or MOI) can differ. These regimes apply to the simulations in the theoretical analyses. Data are for 50 ABM simulations. Data from model output used to produce the figure can be found in https://figshare.com/articles/Figure_data/8079713. ABM, agent-based model; G, generalized immunity; MOI, multiplicity of infection; N, complete neutrality; S, selection. (PDF) [file pbio.3000336.s002.pdf]

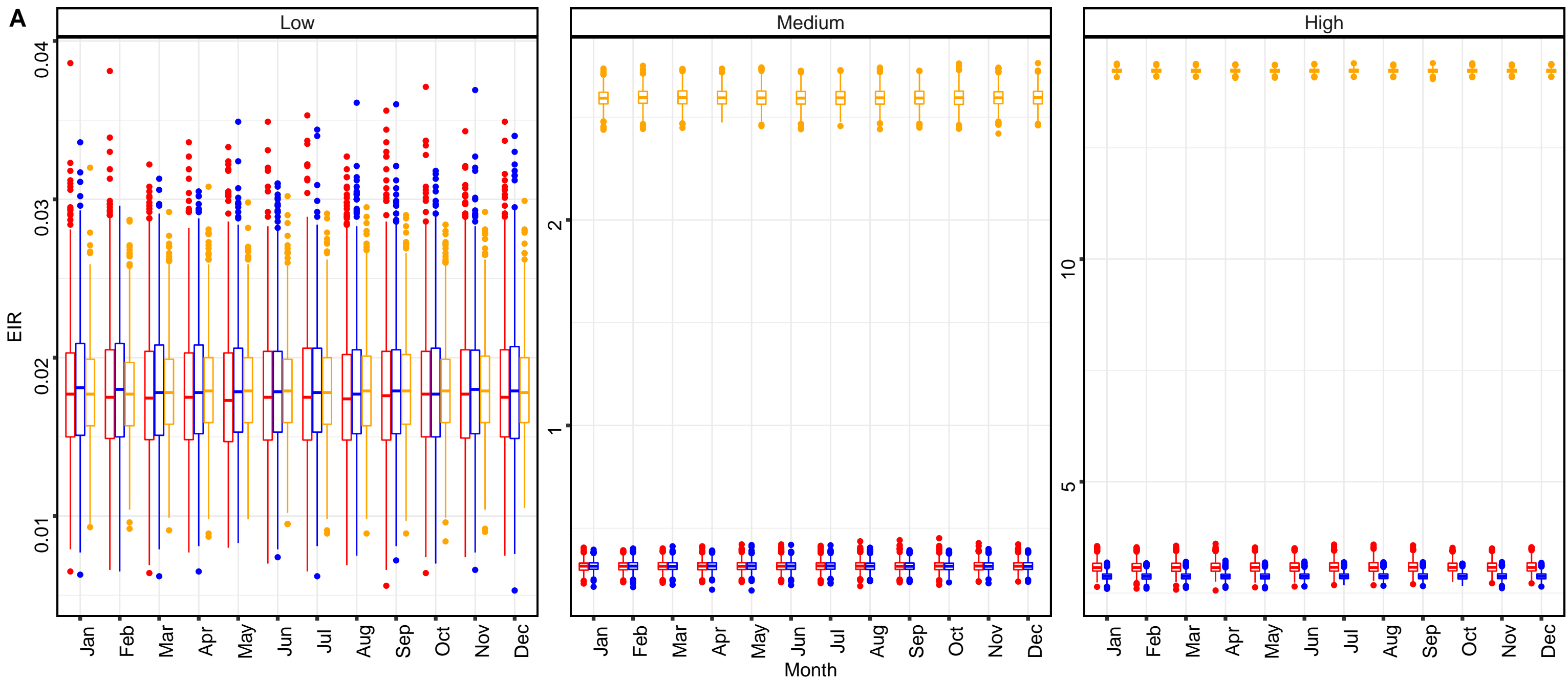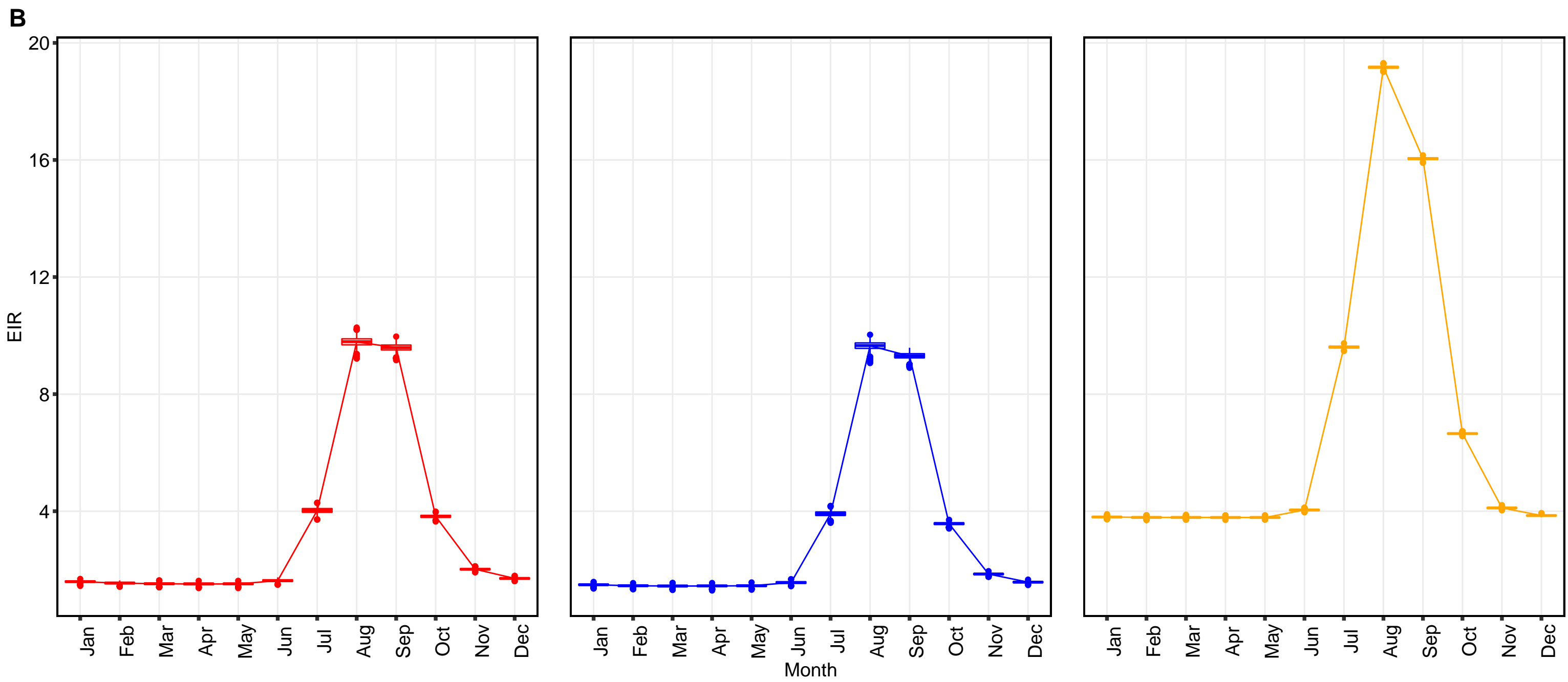

Supplement: S2 Fig — Panels (A) and (B) show the EIR for simulations without and with seasonality, respectively, for the 3 scenarios: immune selection (red), generalized immunity (blue), and complete neutrality (orange). In (A), each panel depicts a different transmission regime. Because there is no seasonality, EIR does not vary across months. In (B), values are for simulations with seasonality in the high transmission regime only, and each panel depicts a different scenario. Data from model output used to produce the figure can be found in https://figshare.com/articles/Figure_data/8079713. ABM, agent-based model; EIR, entomological inoculation rate. (PDF) [file pbio.3000336.s003.pdf]

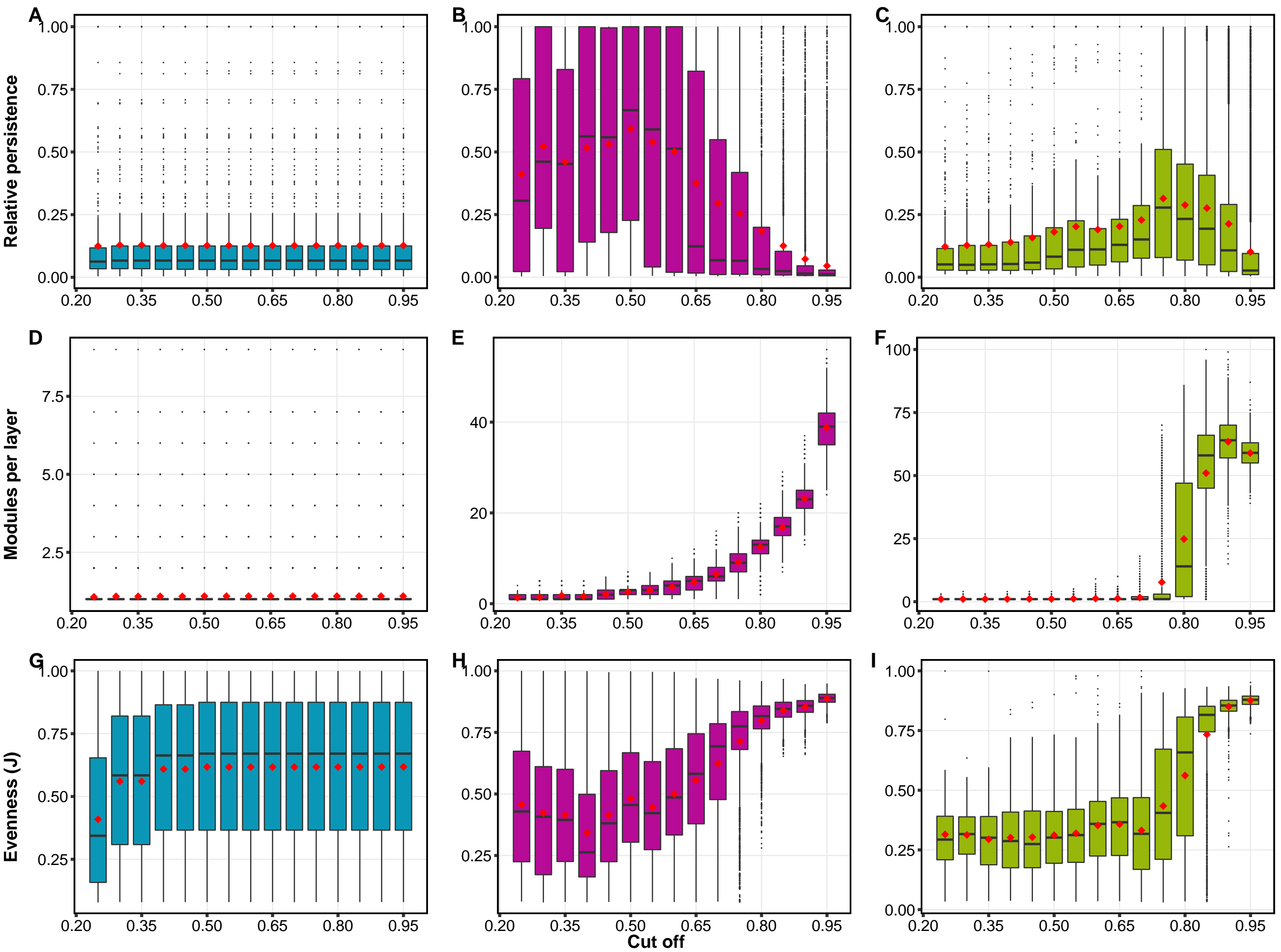

Supplement: S3 Fig — The left, middle, and right columns correspond to the low (teal), medium (purple), and high (green) diversity regimes, respectively, in the immune selection scenario. (A–I) Change in values of three network properties as a function of cutoff percentile (see Methods for details on how we calculated these properties). In each cutoff value, we draw a box plot and mean (red point) for values across 10 simulations of the ABM. We selected cutoff percentiles of 0.3, 0.6, and 0.85 in the low, medium, and high regimes, respectively. The same percentiles were used in the counterpart neutral and generalized immunity model scenarios. Data from model output used to produce the figure can be found in https://figshare.com/articles/Figure_data/8079713. ABM, agent-based model. (PDF) [file pbio.3000336.s004.pdf]

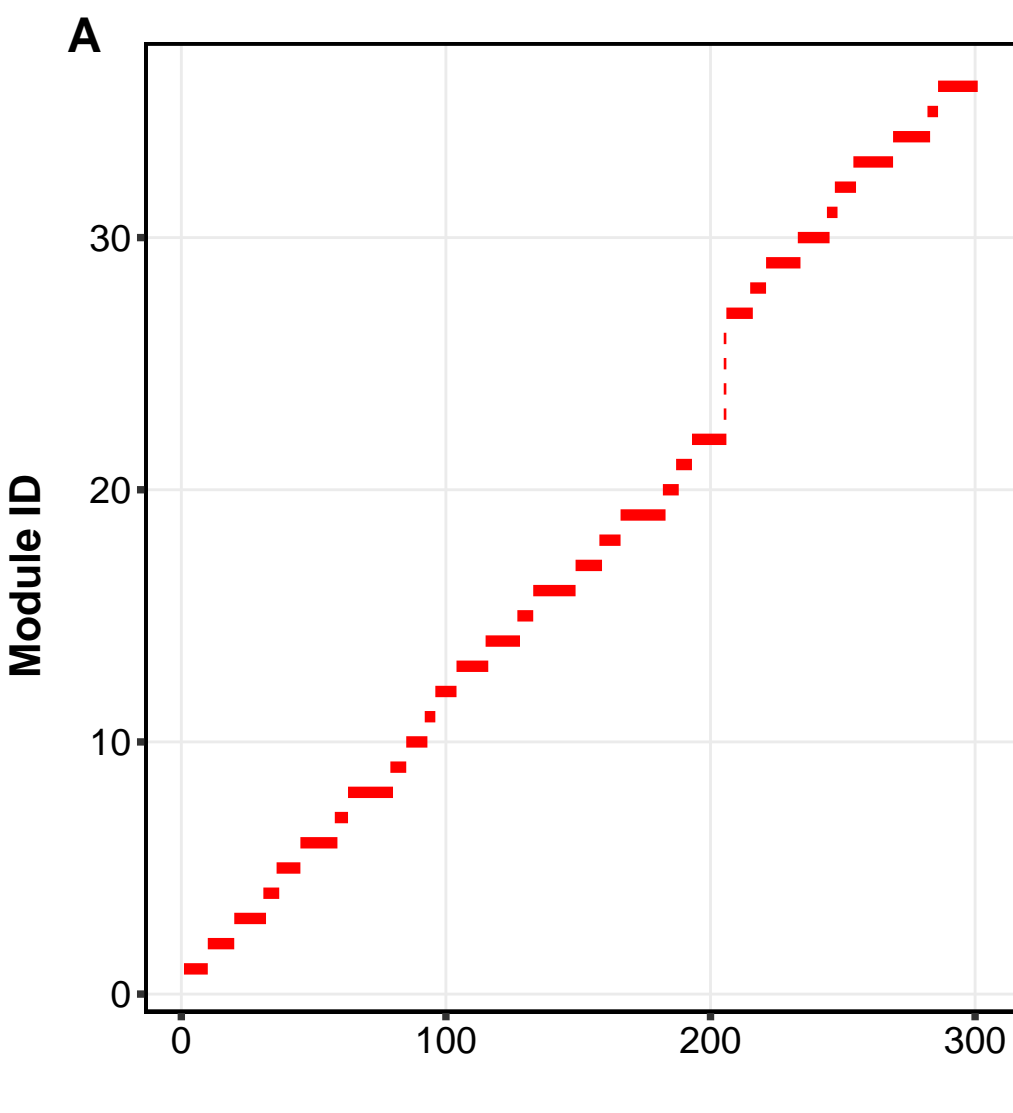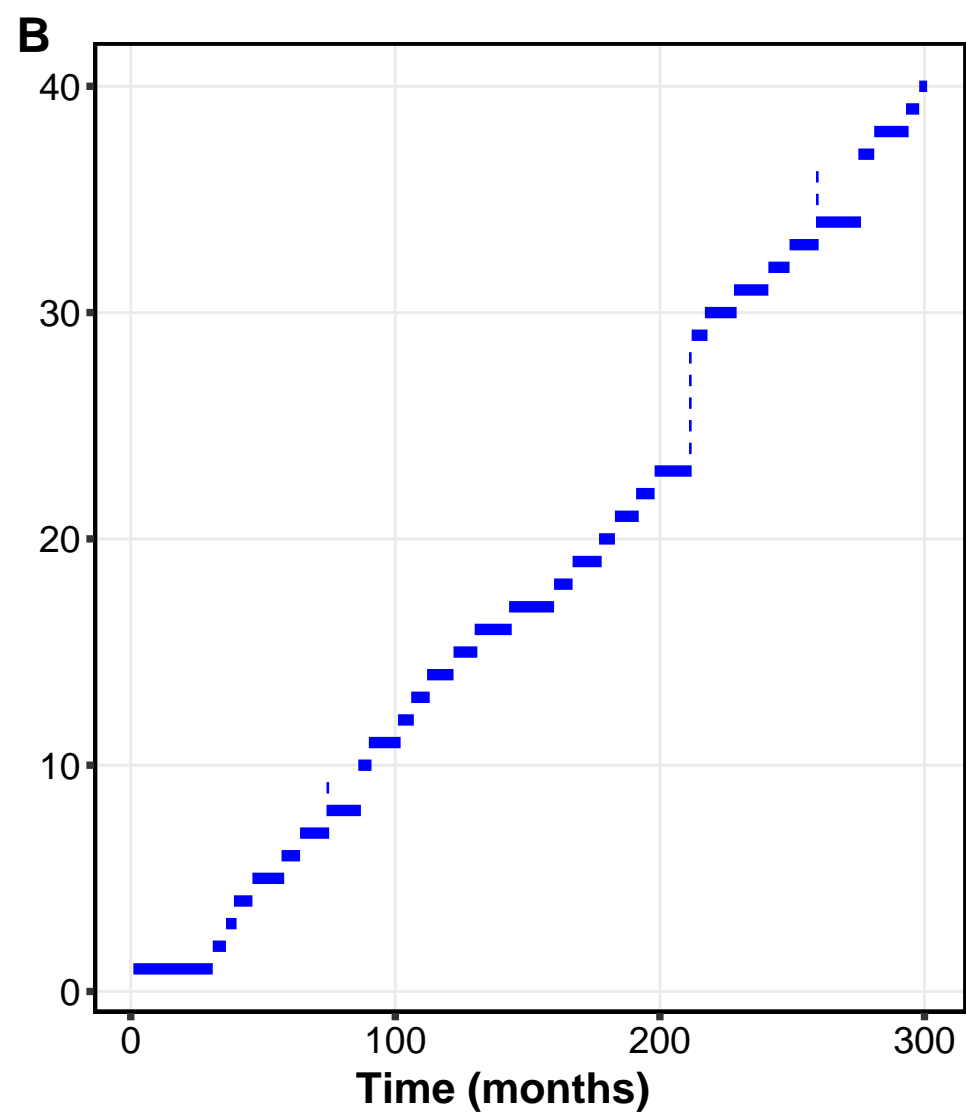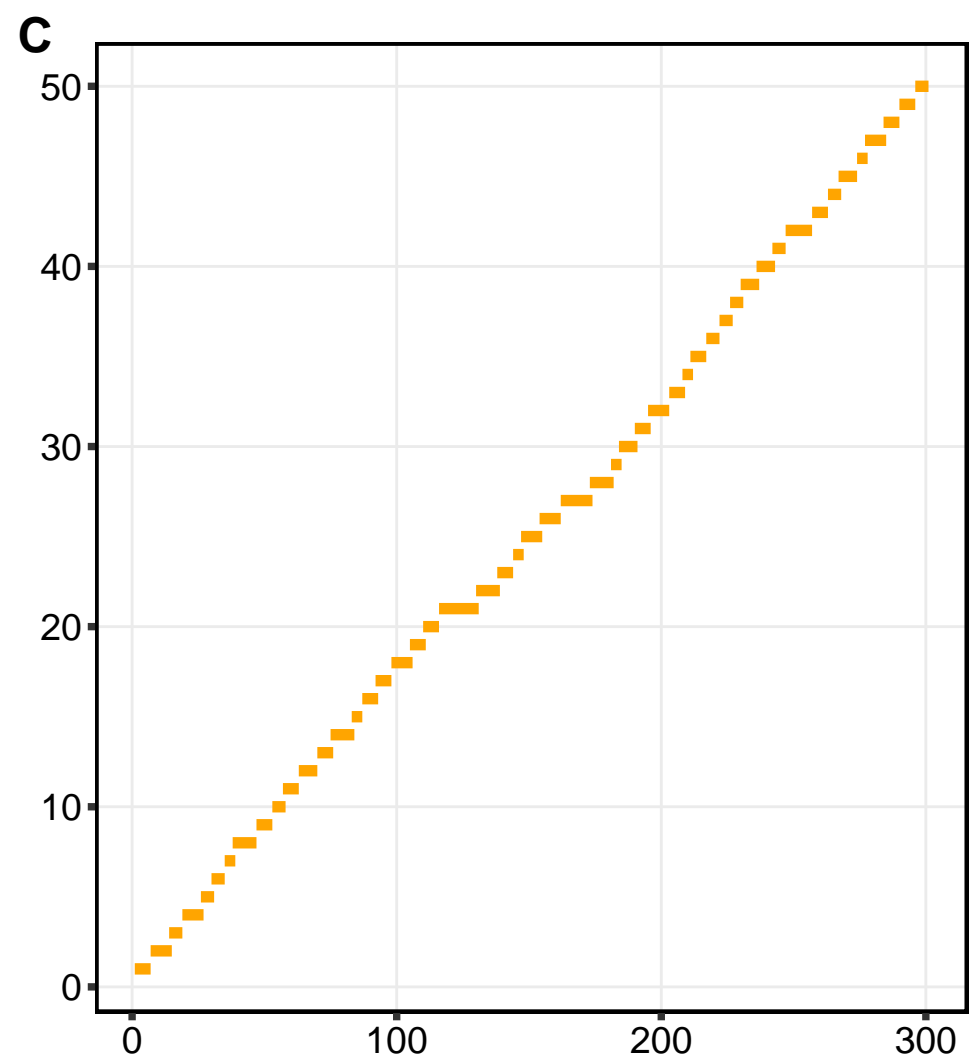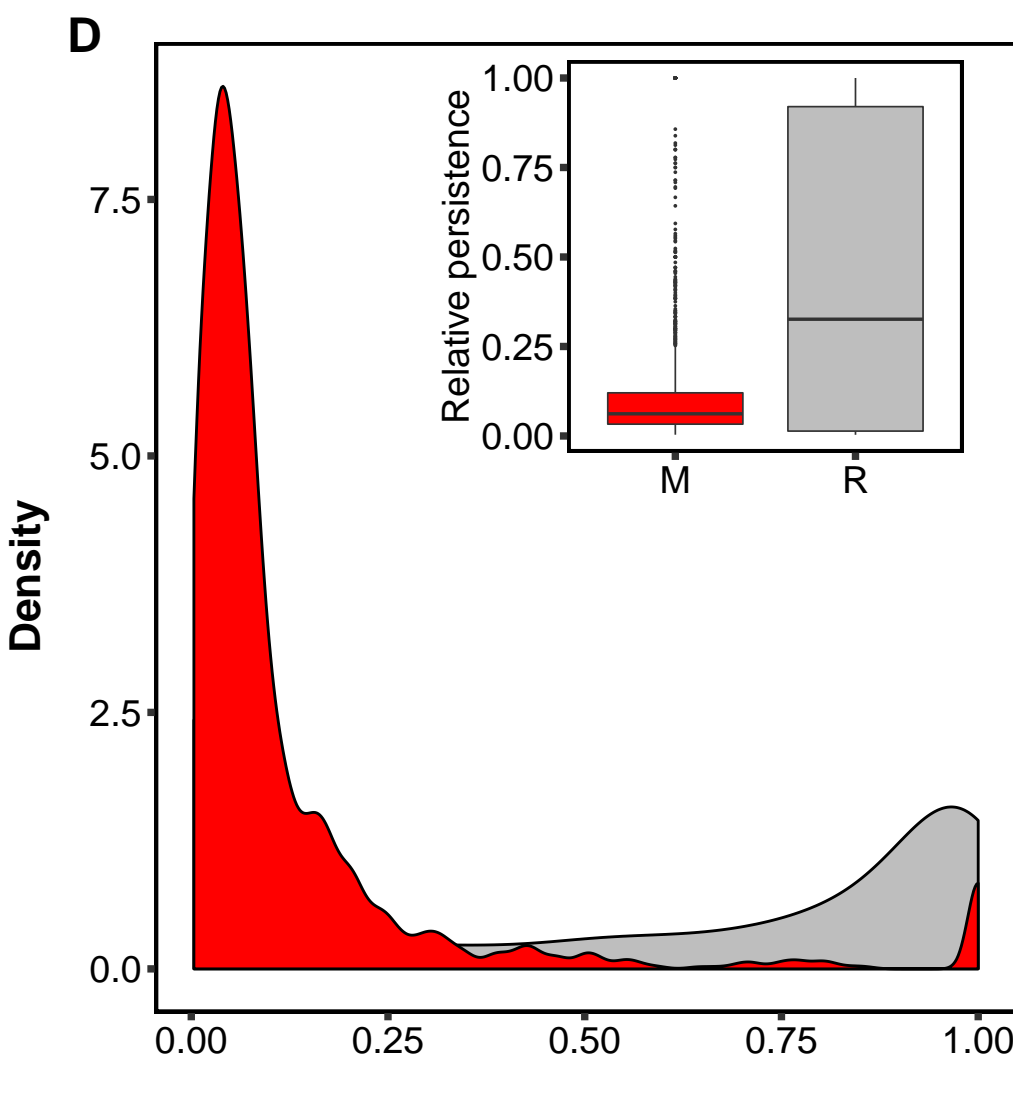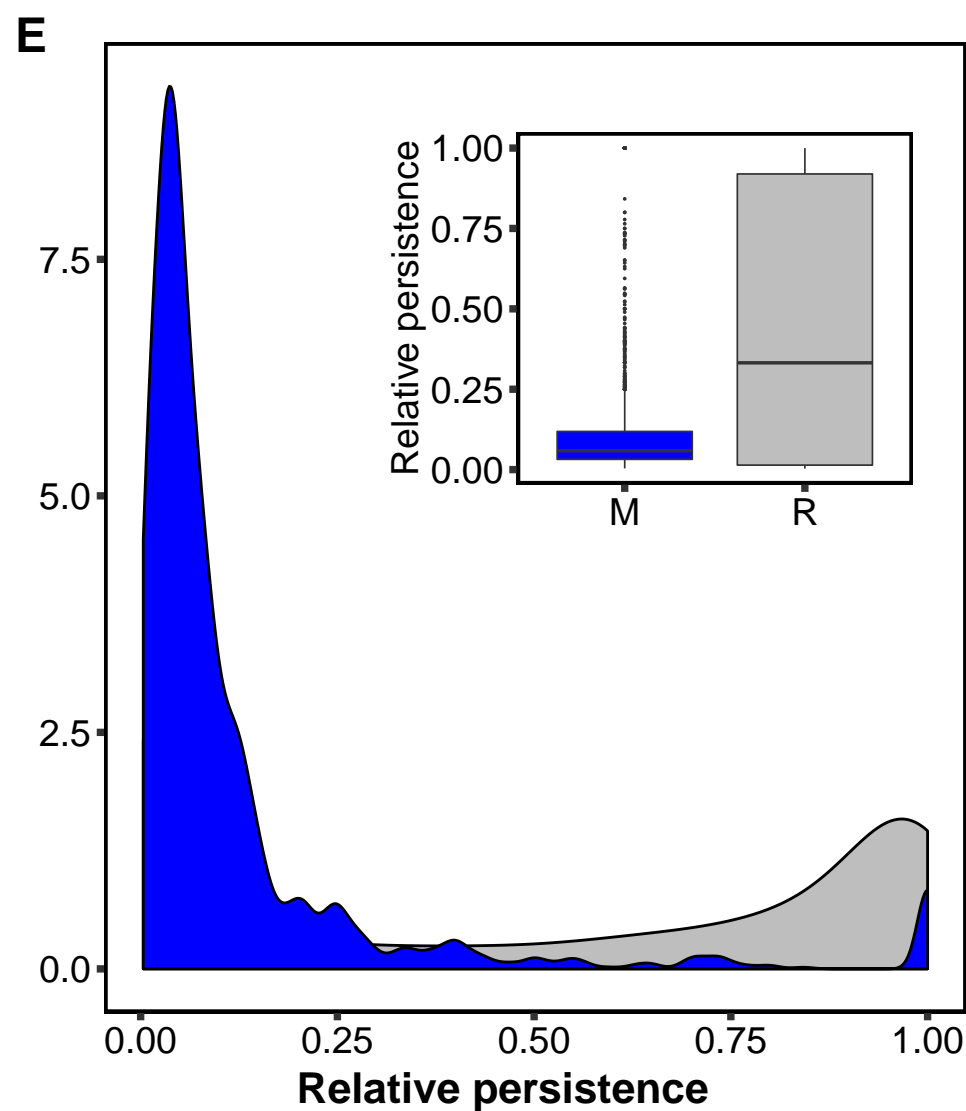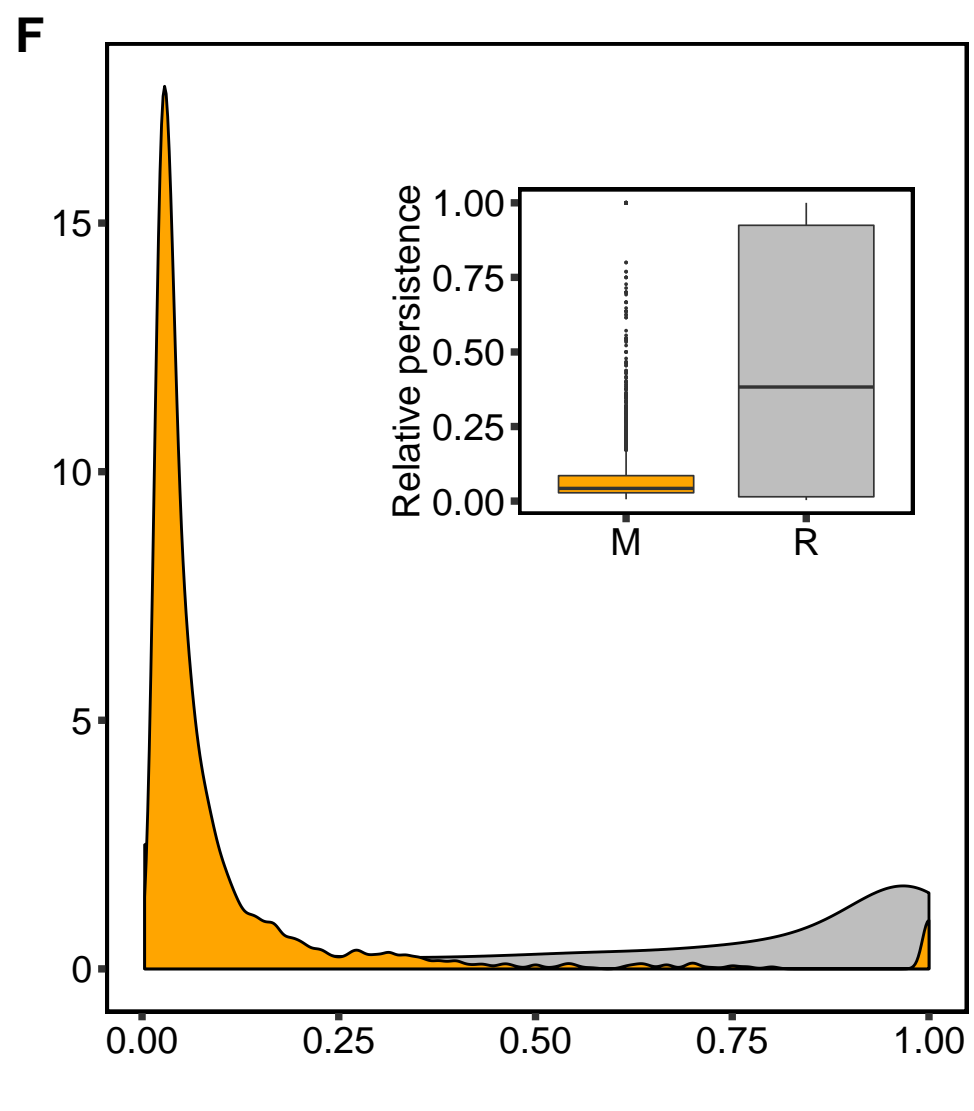

Supplement: S4 Fig — The left, middle, and right columns represent the selection (red), generalized immunity (blue), and neutral (orange) scenarios, respectively. (A,B,C) Example of population structure from one run of the ABM. Each line on the y-axis corresponds to a different module, and its horizontal length depicts its occurrence across layers. Modules are generated and die out. All scenarios are characterized by replacement dynamics whereby one module persists until it is replaced by another, possibly following a short transition period. (D,E,F) These dynamics result in module persistence (colored density plots), which is shorter than that of repertoires (gray density plots) in all scenarios. The insets depict a comparison between the values in the colored and gray density plots using box plots. See Methods for details on how we calculated relative persistence. Results in (D)–(E) are for data pooled across 50 runs of the ABM. Data from model output used to produce the figure can be found in https://figshare.com/articles/Figure_data/8079713. ABM, agent-based model. (PDF) [file pbio.3000336.s005.pdf]

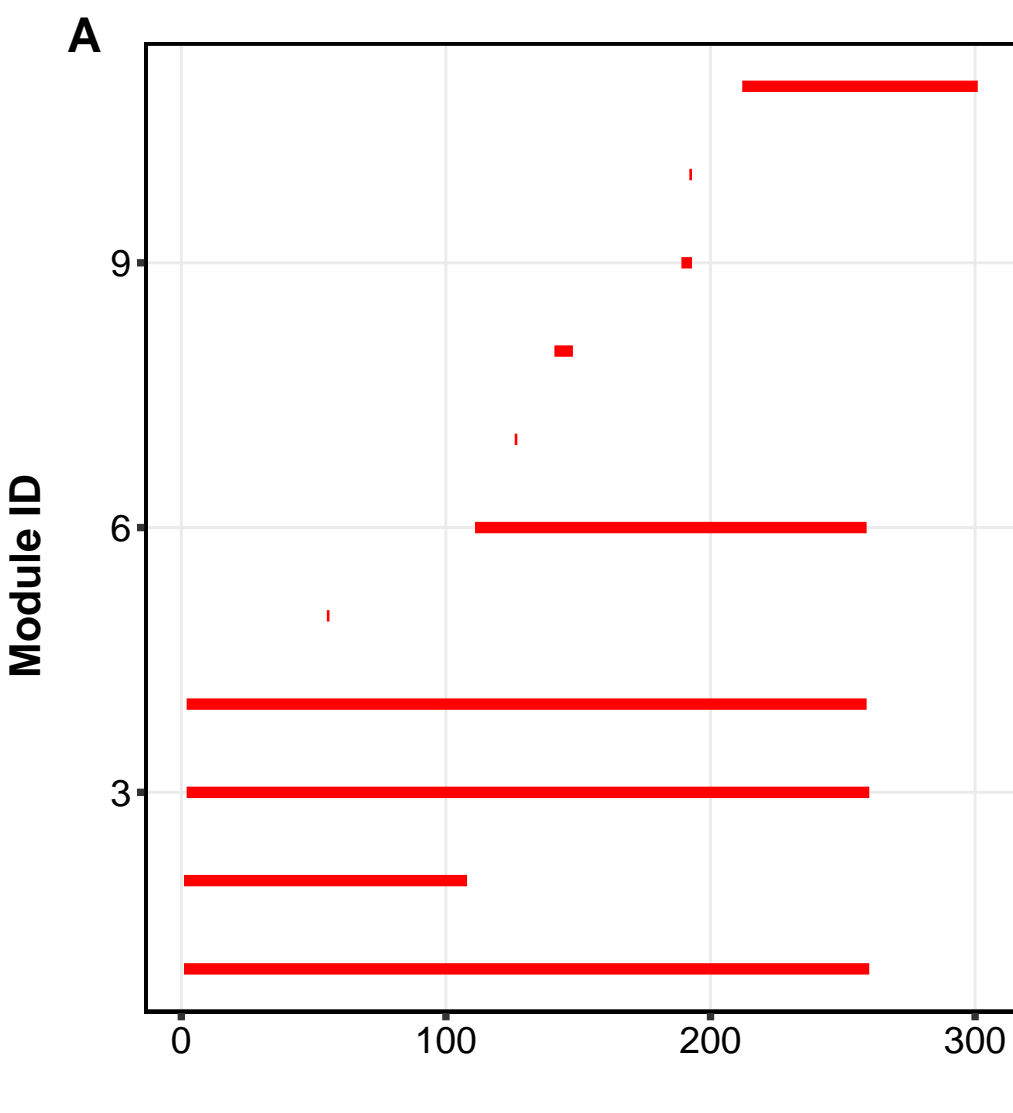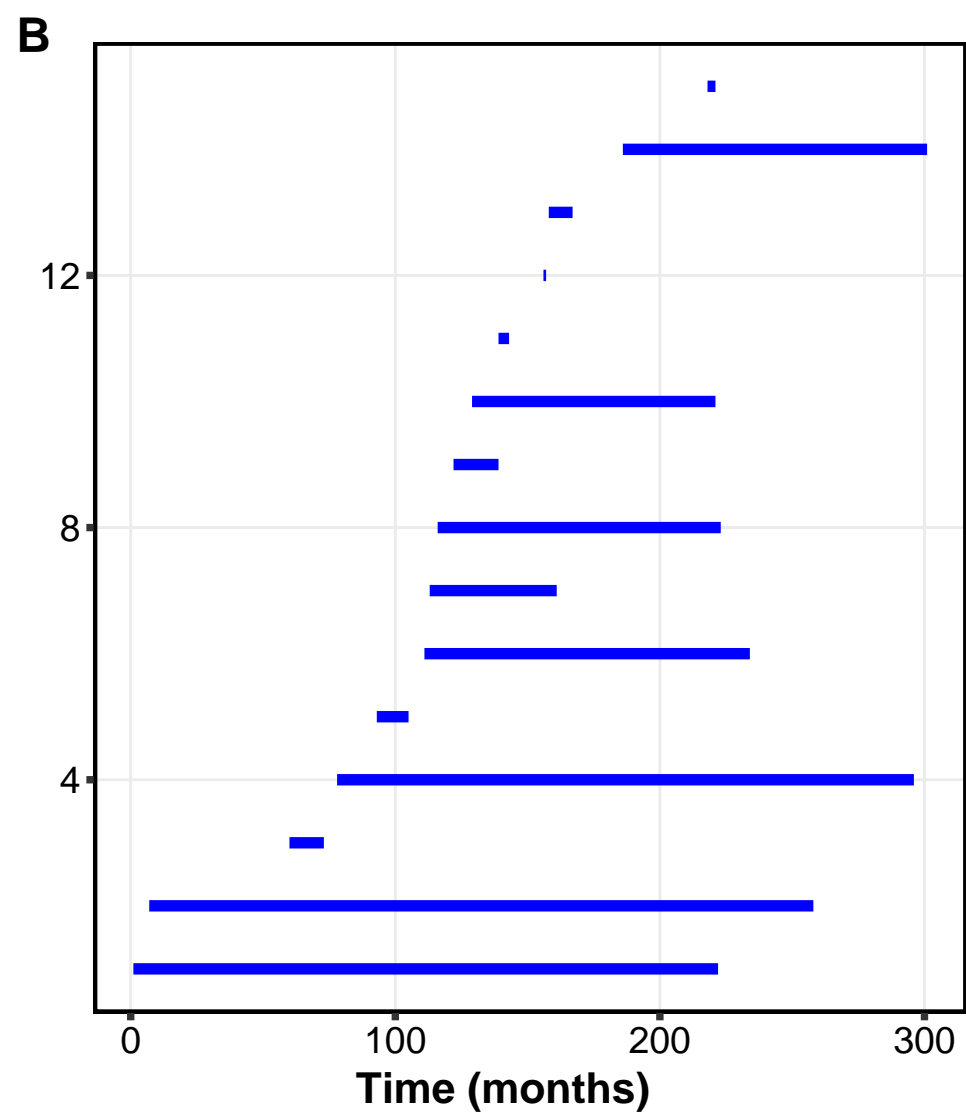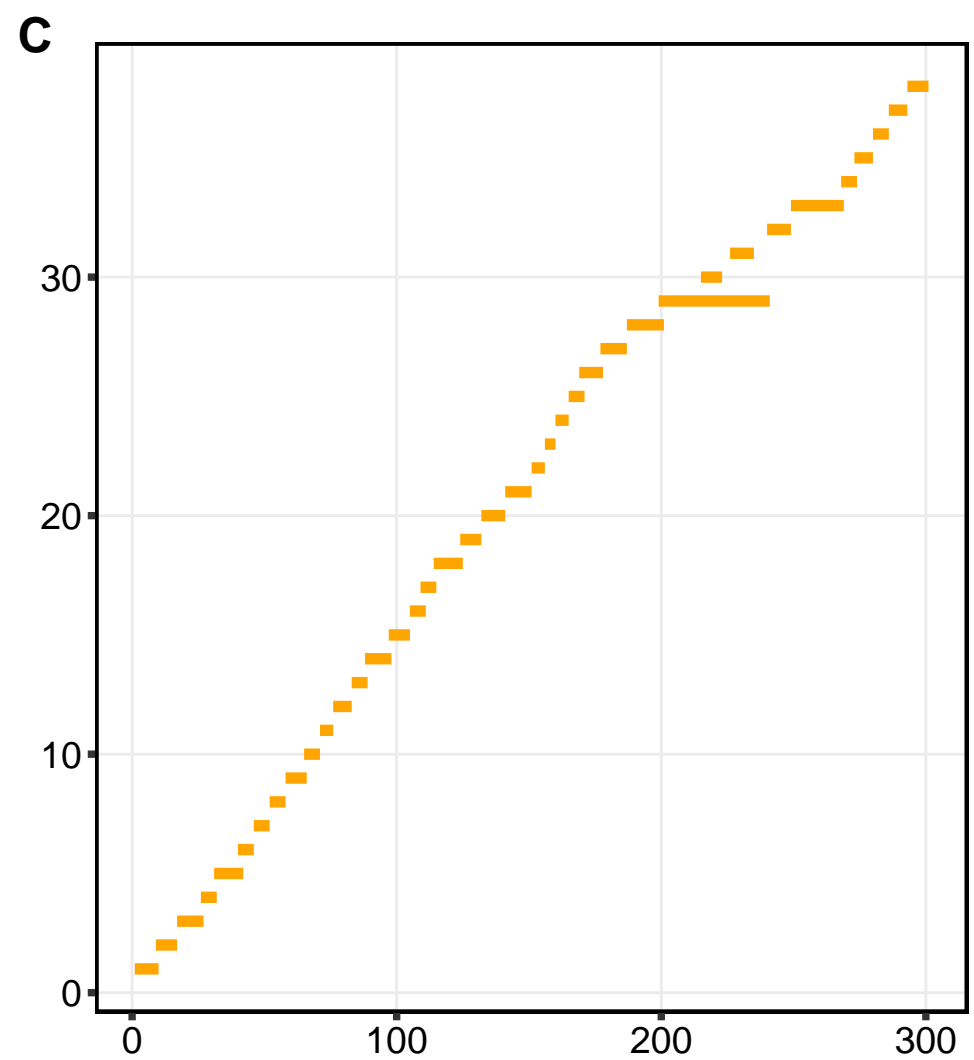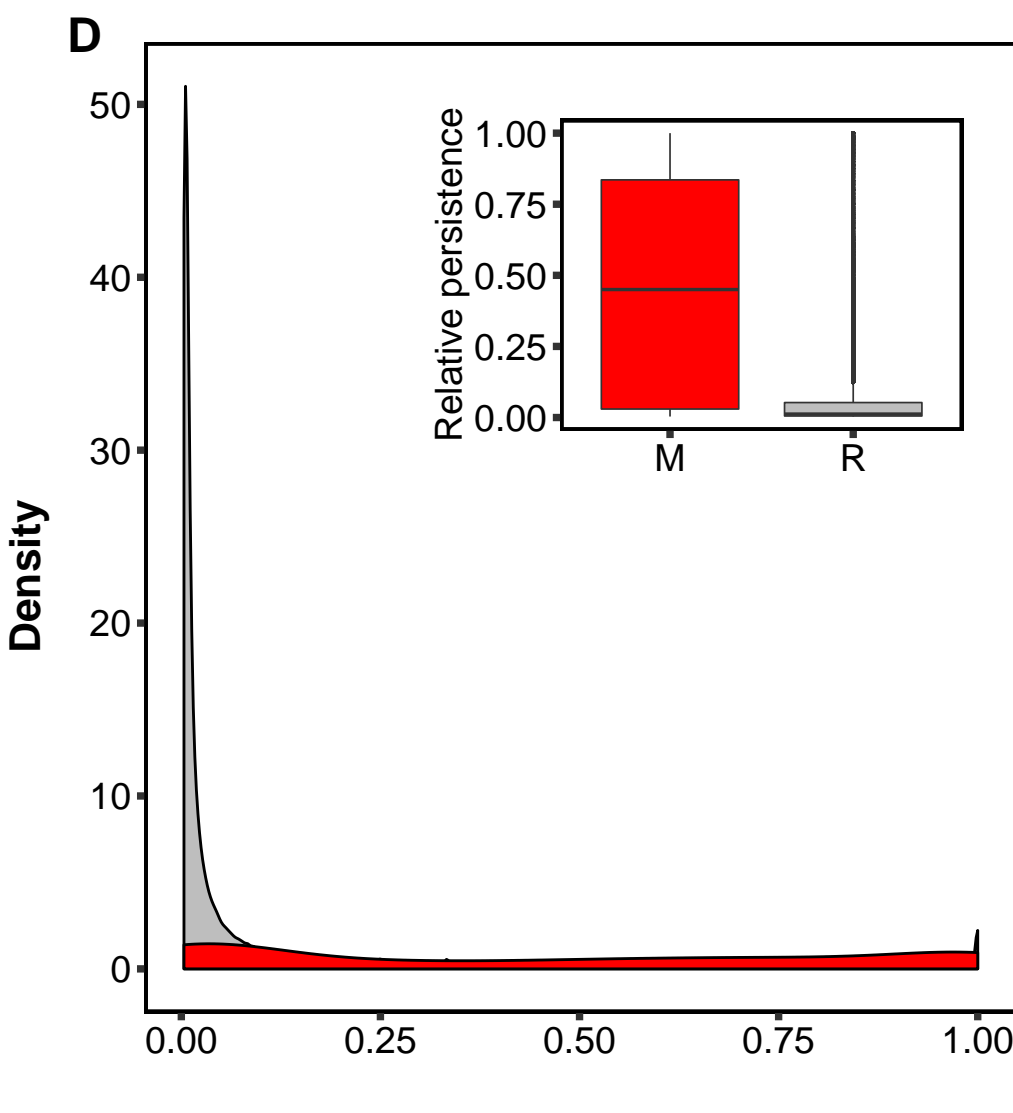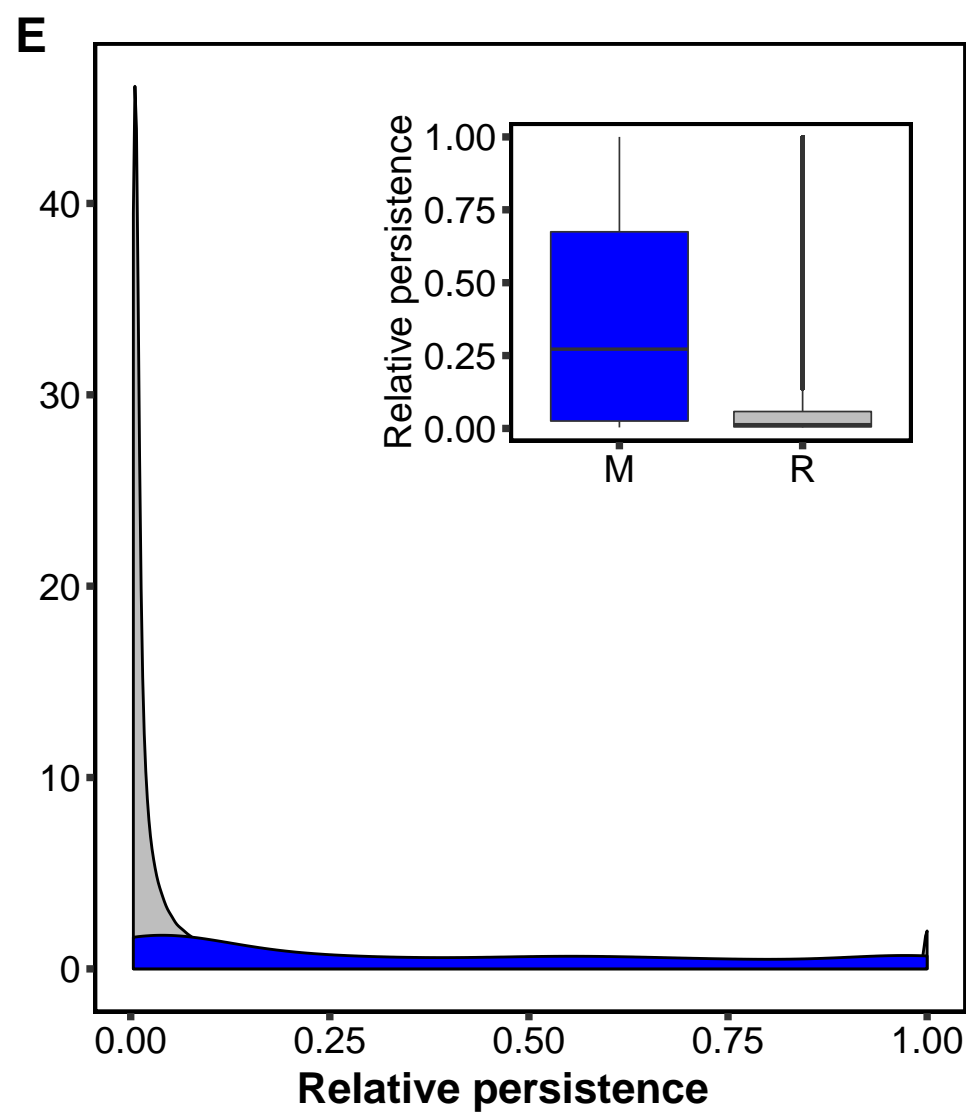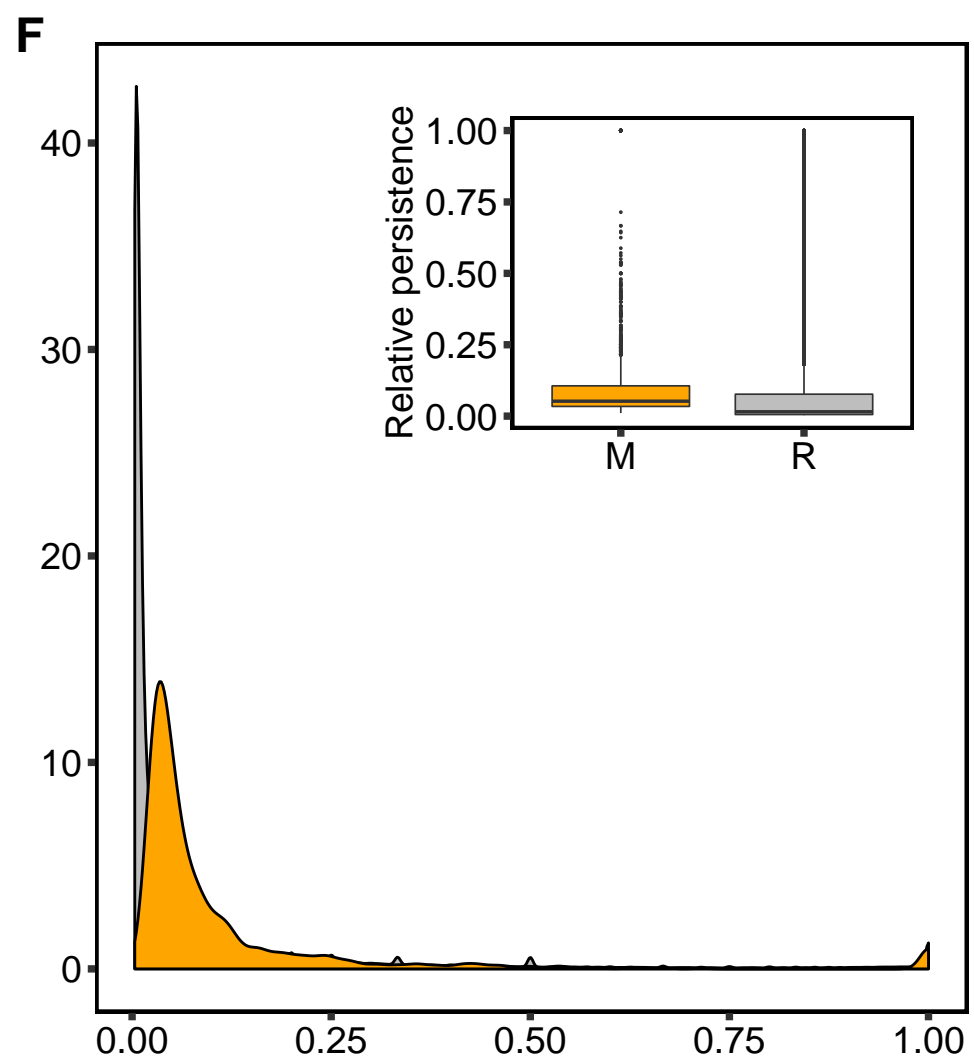

Supplement: S5 Fig — The left, middle, and right columns represent the selection (red), generalized immunity (blue), and neutral (orange) scenarios, respectively. (A,B,C) Example of population structure from one run of the ABM. Each line on the y-axis corresponds to a different module, and its horizontal length depicts its occurrence across layers. Modules are generated and die out. Under complete neutrality, the persistence of repertoires is on the same scale as that of modules, as expected (F). However, there is no apparent difference in population structure and patterns of relative persistence between immune selection and generalized immunity (D,E) (see results for low- and medium-diversity regimes in S1 Text for details). In panels (D)–(F), the insets depict a comparison between the values in the colored and gray density plots using box plots. See Methods for details on how we calculated relative persistence. Results in (D)–(E) are for data pooled across 50 runs of the ABM. Data from model output used to produce the figure can be found in https://figshare.com/articles/Figure_data/8079713. ABM, agent-based model. (PDF) [file pbio.3000336.s006.pdf]

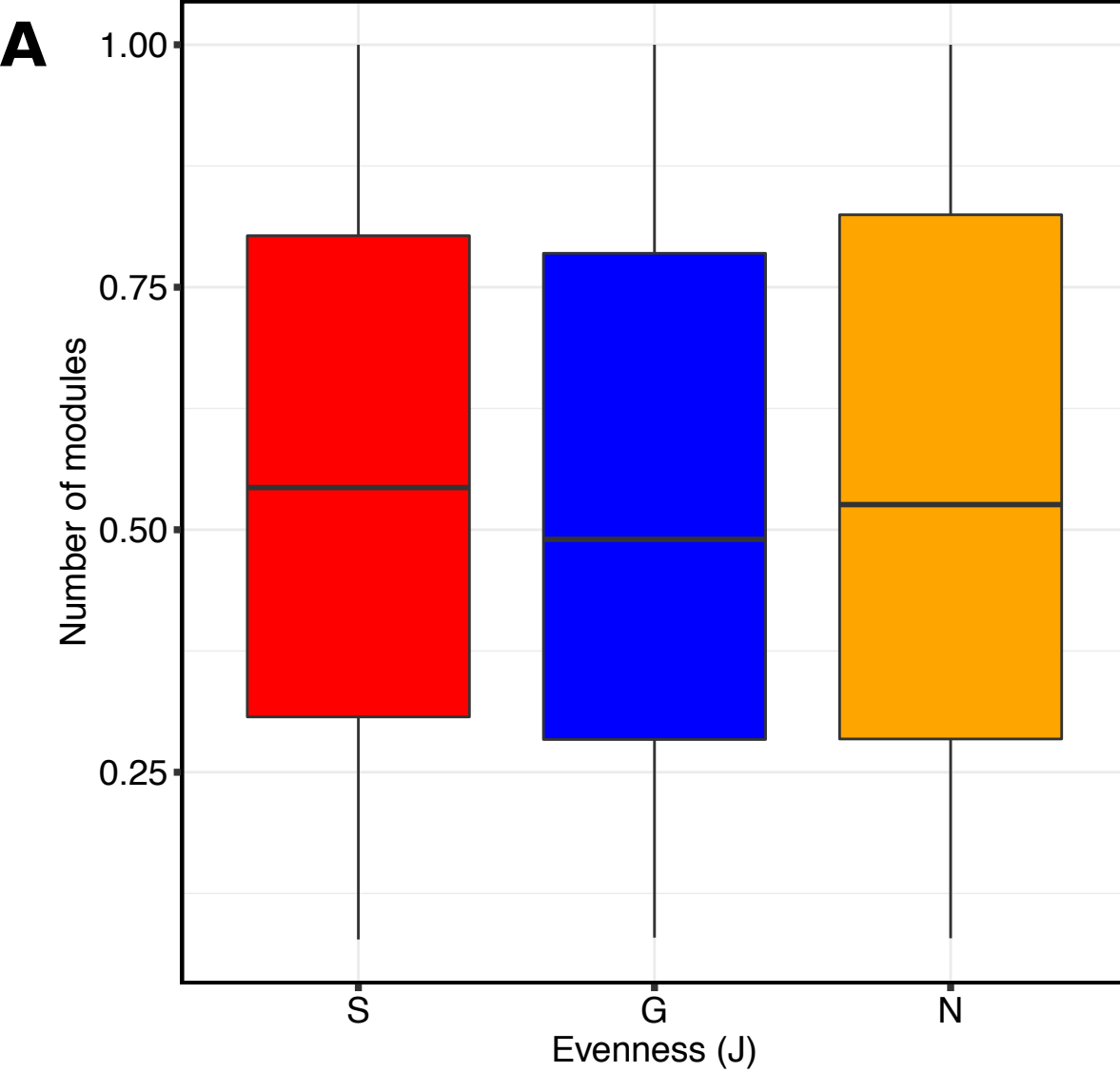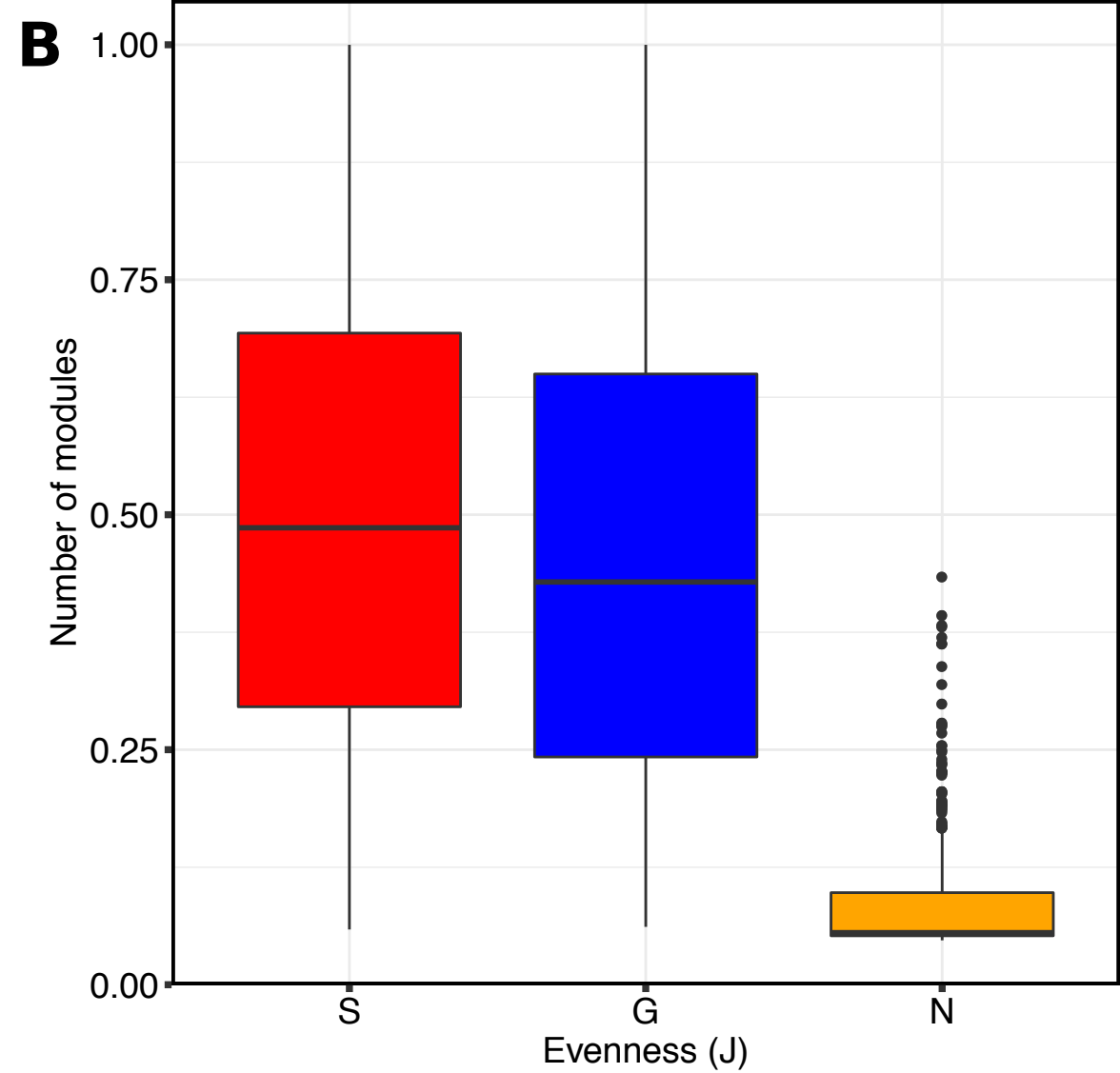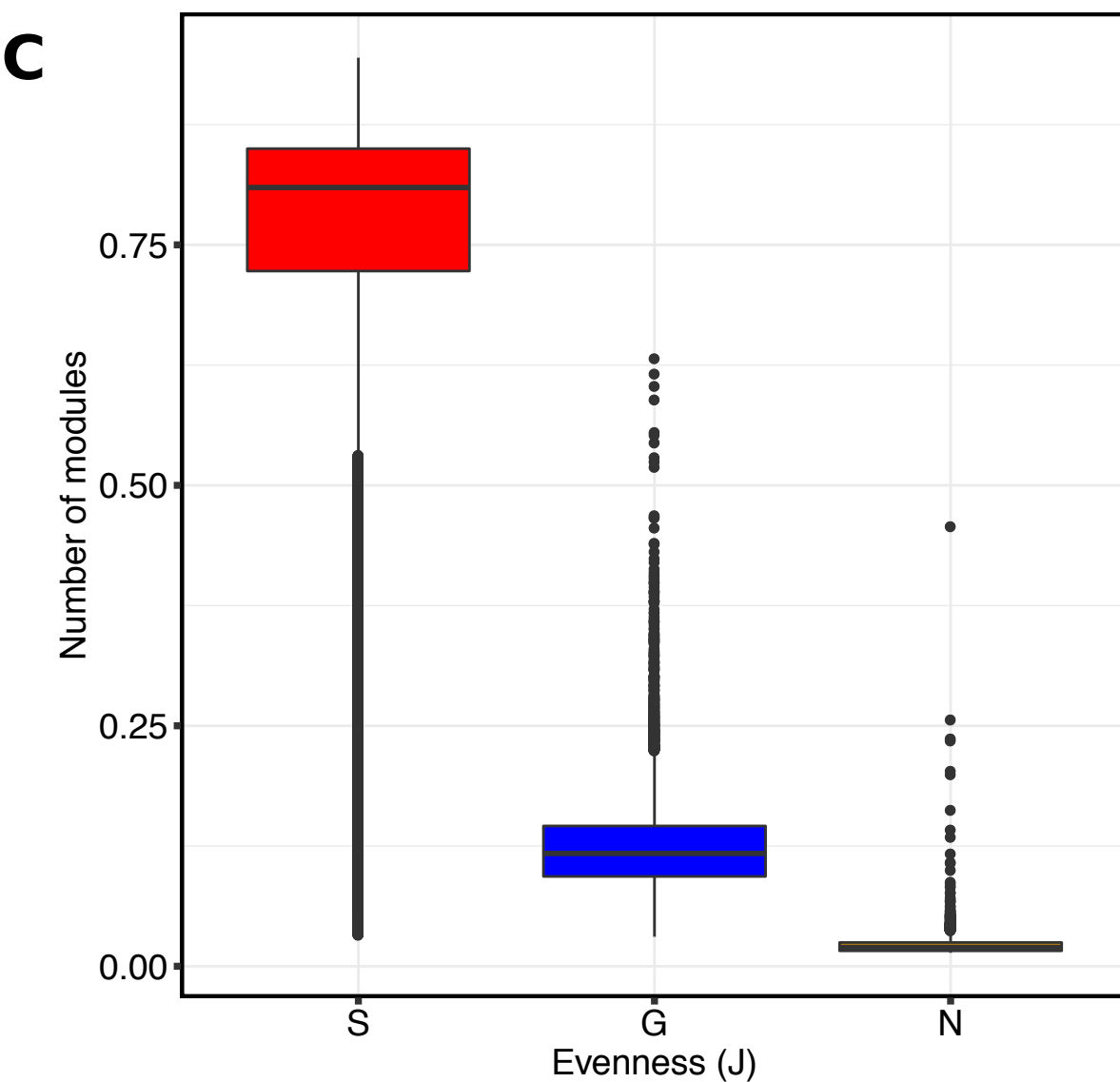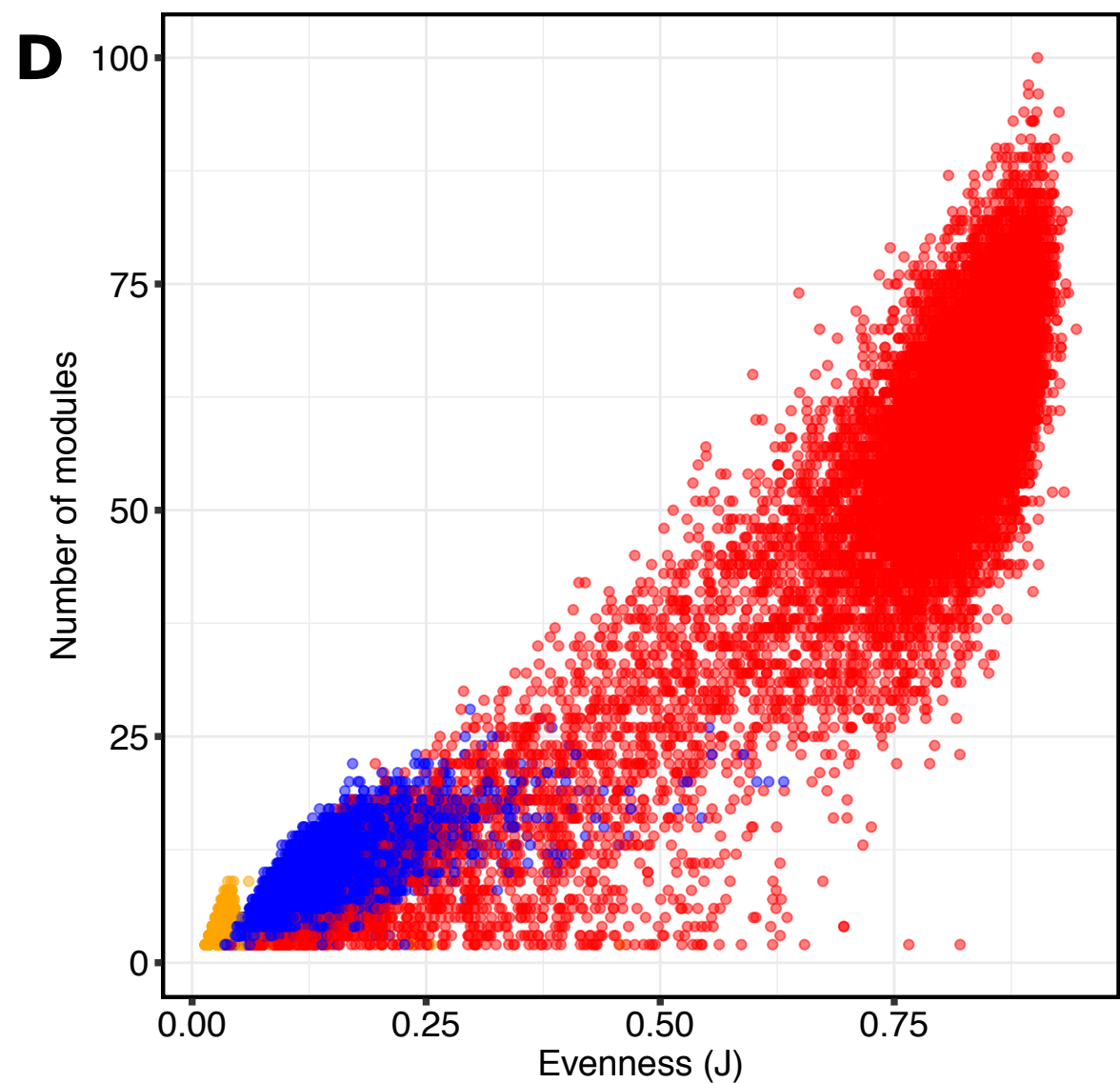

Supplement: S6 Fig — Limiting similarity resulting from selection should promote an even distribution of repertoires across modules. We calculated the evenness J in each layer as J = H′/Hmax [55], where H′ is Shannon's diversity and Hmax is the maximum diversity possible, obtained when all modules have the same size [55]. J varies between 0 and 1, where 1 indicates a perfect distribution of module sizes such that repertoires are at the “limit” of their limiting similarity because each repertoire is as different from any other as possible. We compare between scenarios using box plots for the low- (A), medium- (B), and high-diversity regimes (C). Because immune selection does not operate in the low-diversity regime, the evenness is comparable across scenarios. By contrast to high diversity, competition under medium diversity is not strong enough to generate patterns that distinguish between immune selection and generalized immunity. (D) For high diversity, evenness is higher when there are more modules in a layer. Results are for data pooled across 50 runs of the ABM. Data from model output used to produce the figure can be found in https://figshare.com/articles/Figure_data/8079713. ABM, agent-based model; G, generalized immunity; N, complete neutrality; S, Immune selection. (PDF) [file pbio.3000336.s007.pdf]

**A**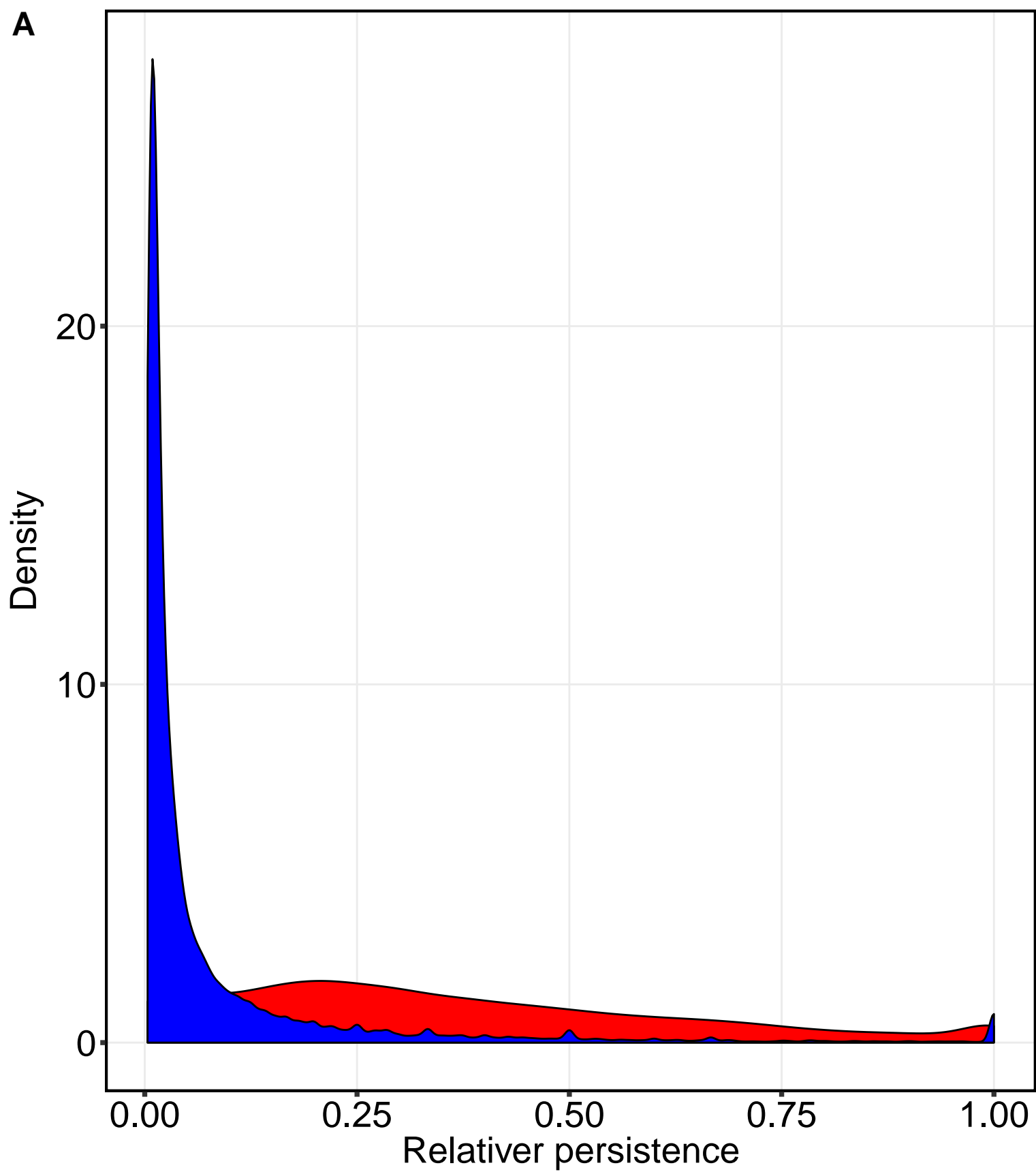**B**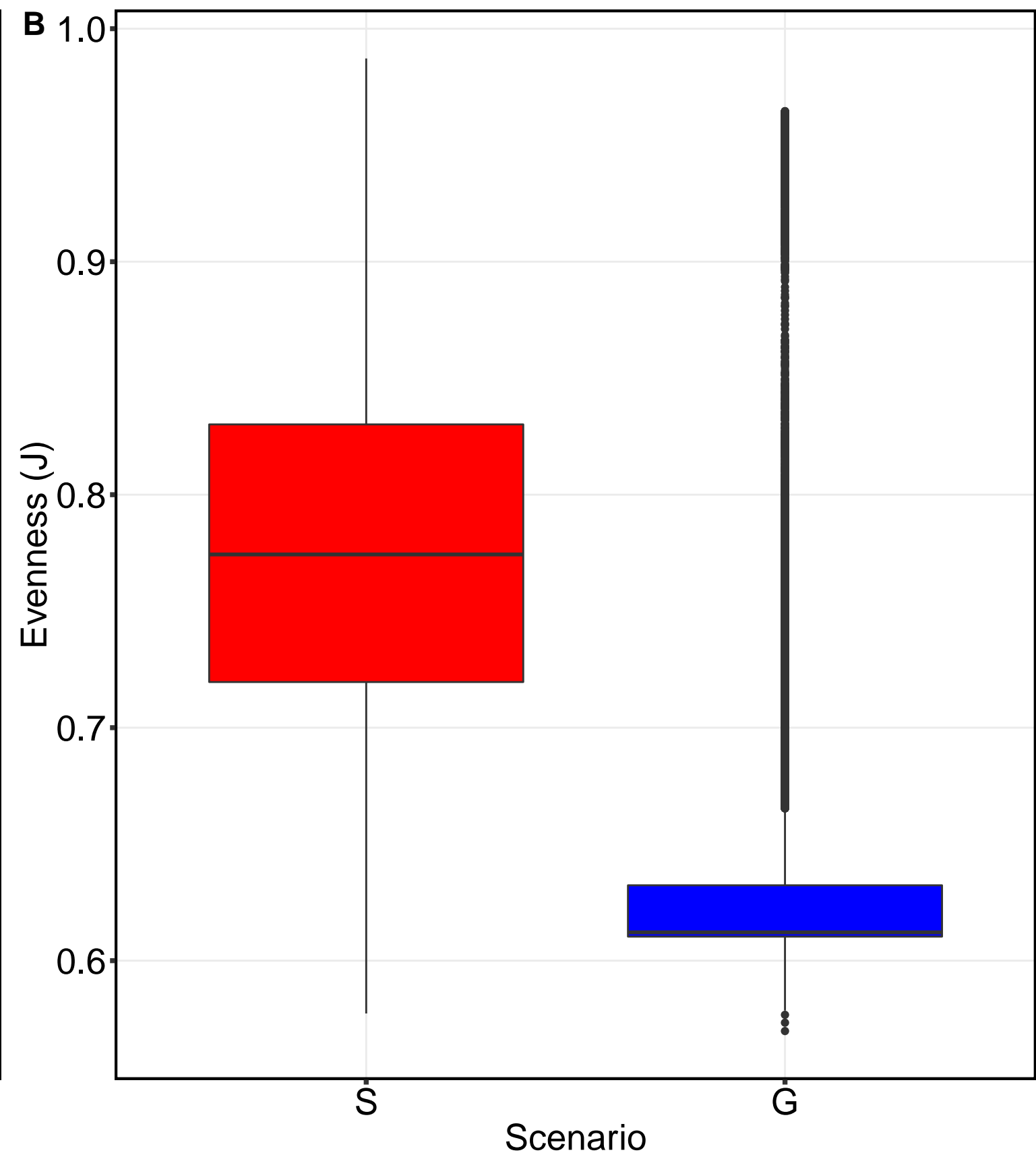

Supplement: S7 Fig — We repeat the analysis of the theoretical part of the main text using 112 simulations for the immune selection (red) and generalized immunity (blue) scenarios. These 112 simulations encompass variation in three key parameters of the ABM. We find the same qualitative results for the differences between the two scenarios as in the main text. Specifically, the relative persistence of modules (𝒫) is longer (A), and the evenness in module size is higher (B) under immune selection compared to generalized immunity. Data from model output used to produce the figure can be found in https://figshare.com/articles/Figure_data/8079713. ABM, agent-based model. (PDF) [file pbio.3000336.s008.pdf]

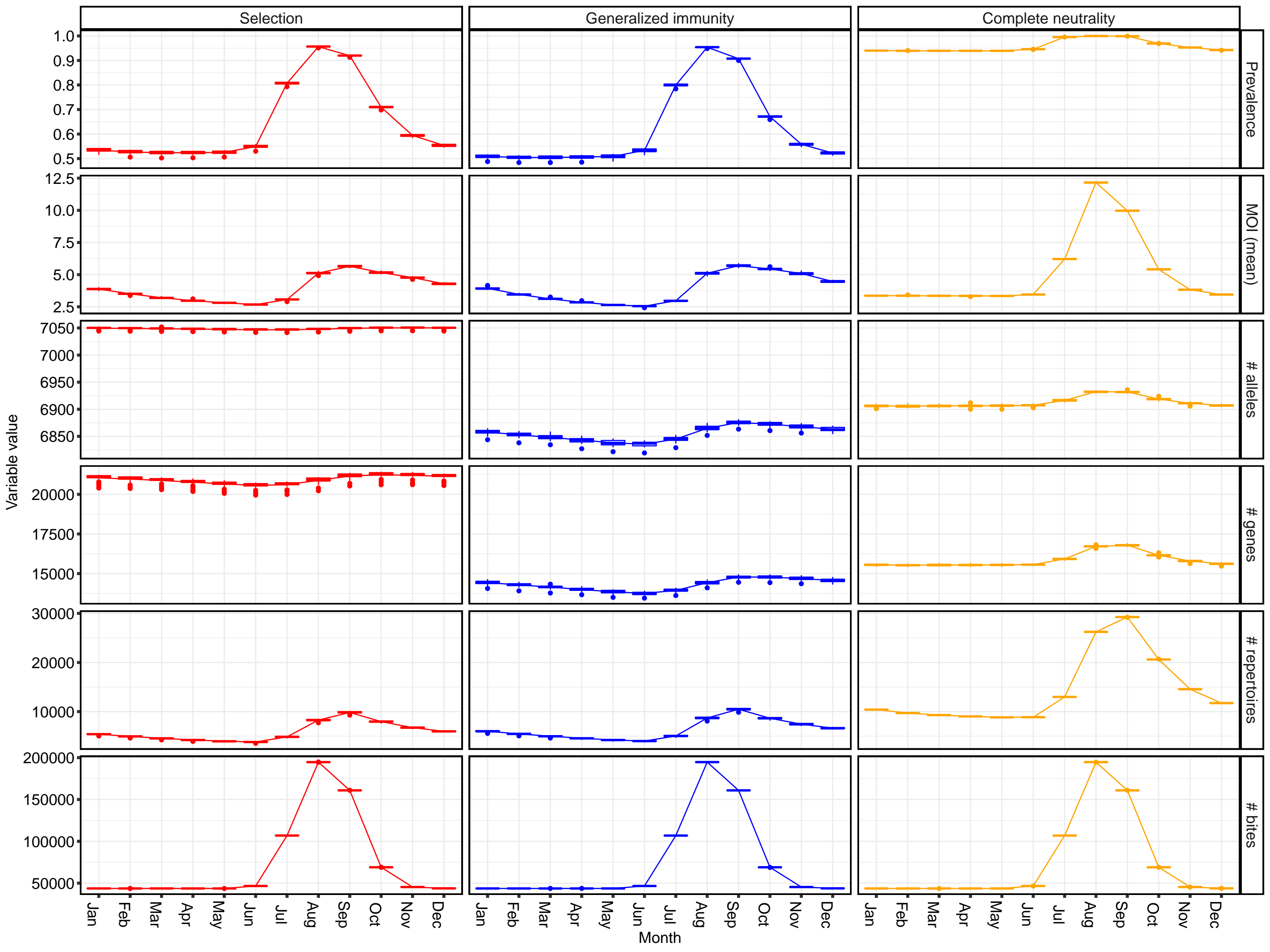

Supplement: S8 Fig — The left, middle, and right columns represent the selection, generalized immunity, and complete neutrality scenarios, respectively. Each row depicts one parameter relevant for epidemiology or genetic diversity. This regime is for the baseline simulations of seasonality, with G = 35,000, used to confirm that our nonseasonal theoretical results hold. Data are for 50 ABM simulations. Data from model output used to produce the figure can be found in https://figshare.com/articles/Figure_data/8079713. ABM, agent-based model. (PDF) [file pbio.3000336.s009.pdf]

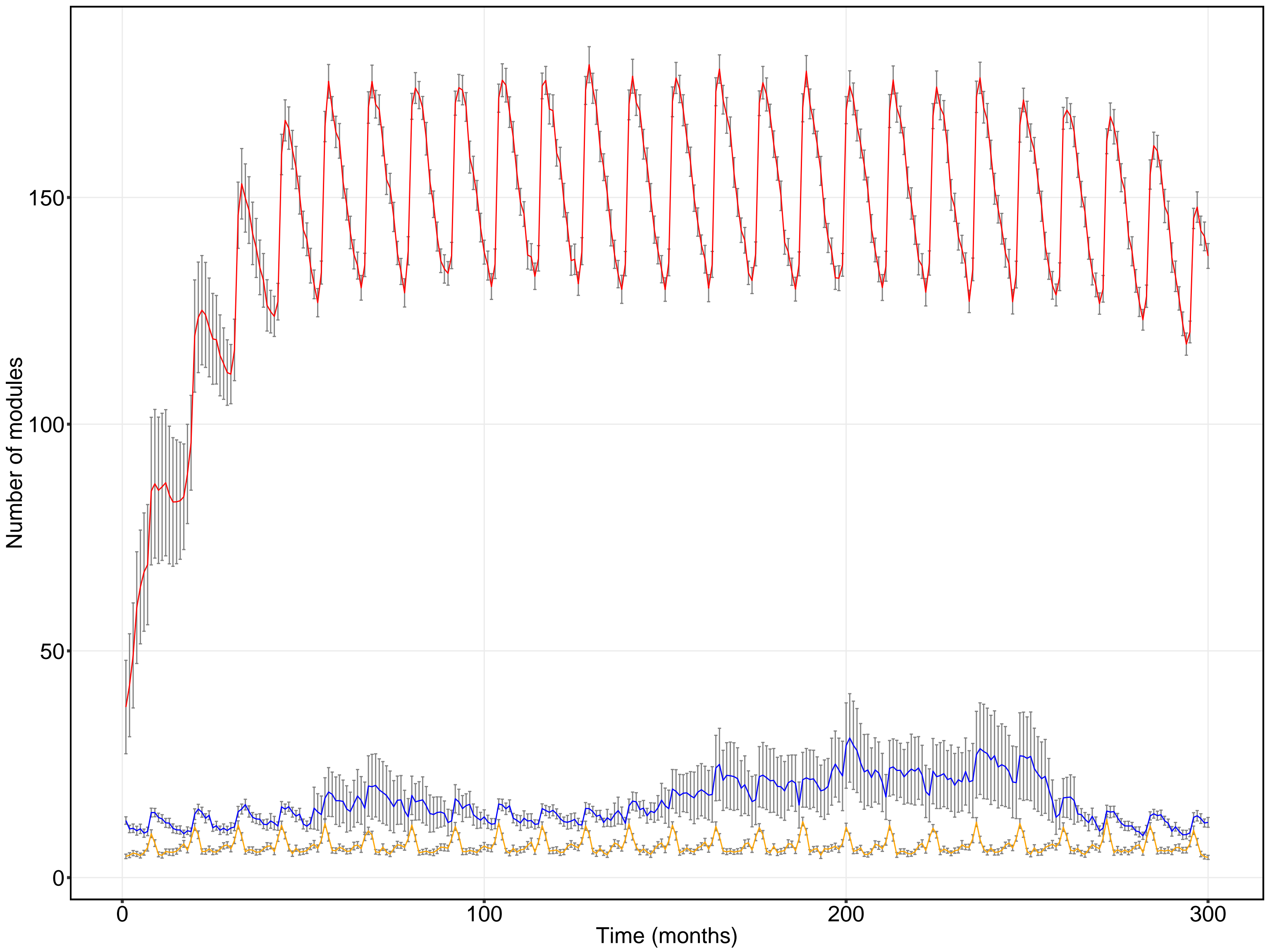

Supplement: S9 Fig — Seasonality promotes the extinction of repertoires in the dry season and the generation of new ones in the wet season. This bottleneck causes a decrease in the creation of new niches, translating to a decrease in the number of modules in the dry season. Data are pooled across 50 runs of the ABM. Error bars represent 95% confidence intervals. Red: immune selection; blue: generalized immunity; orange: complete neutrality. Data from model output used to produce the figure can be found in https://figshare.com/articles/Figure_data/8079713. ABM, agent-based model. (PDF) [file pbio.3000336.s010.pdf]

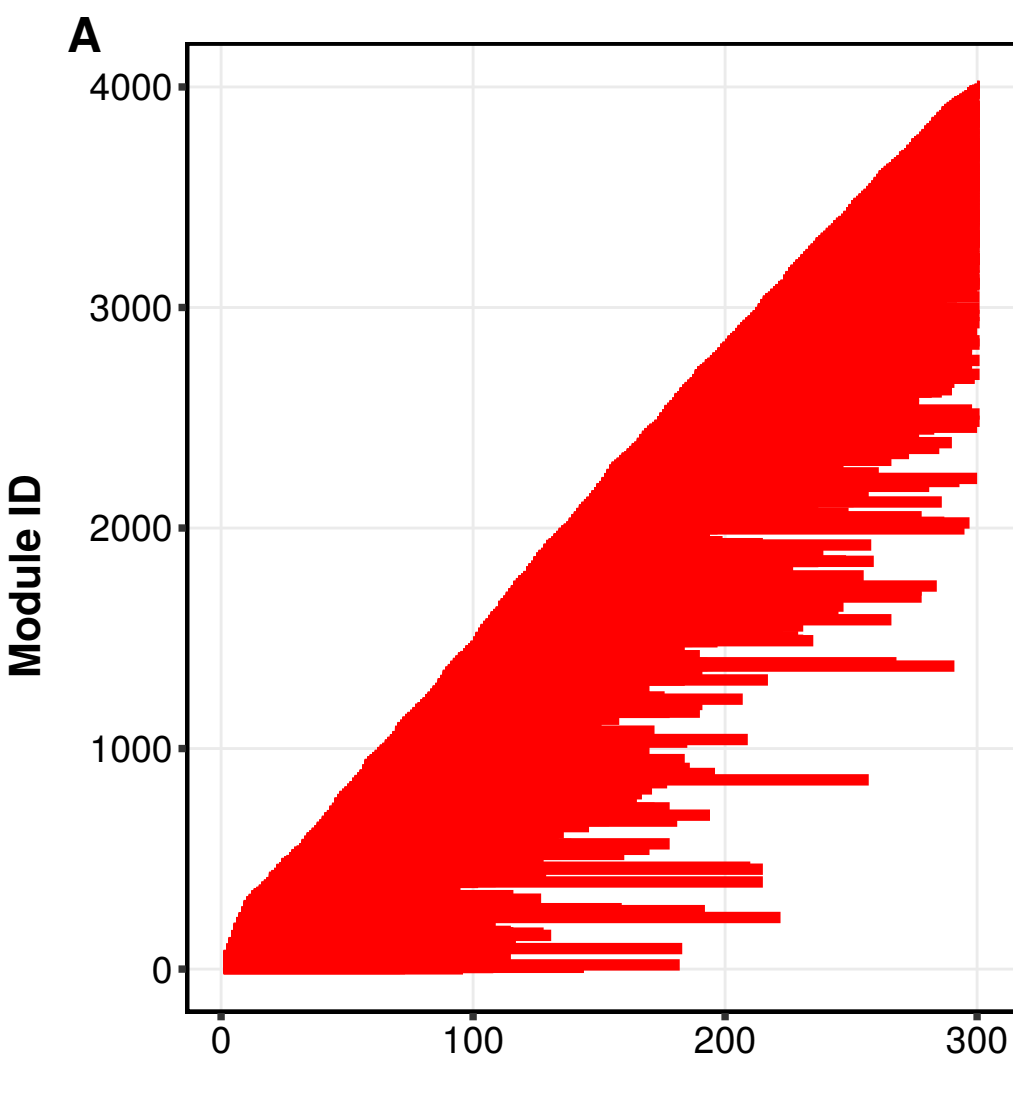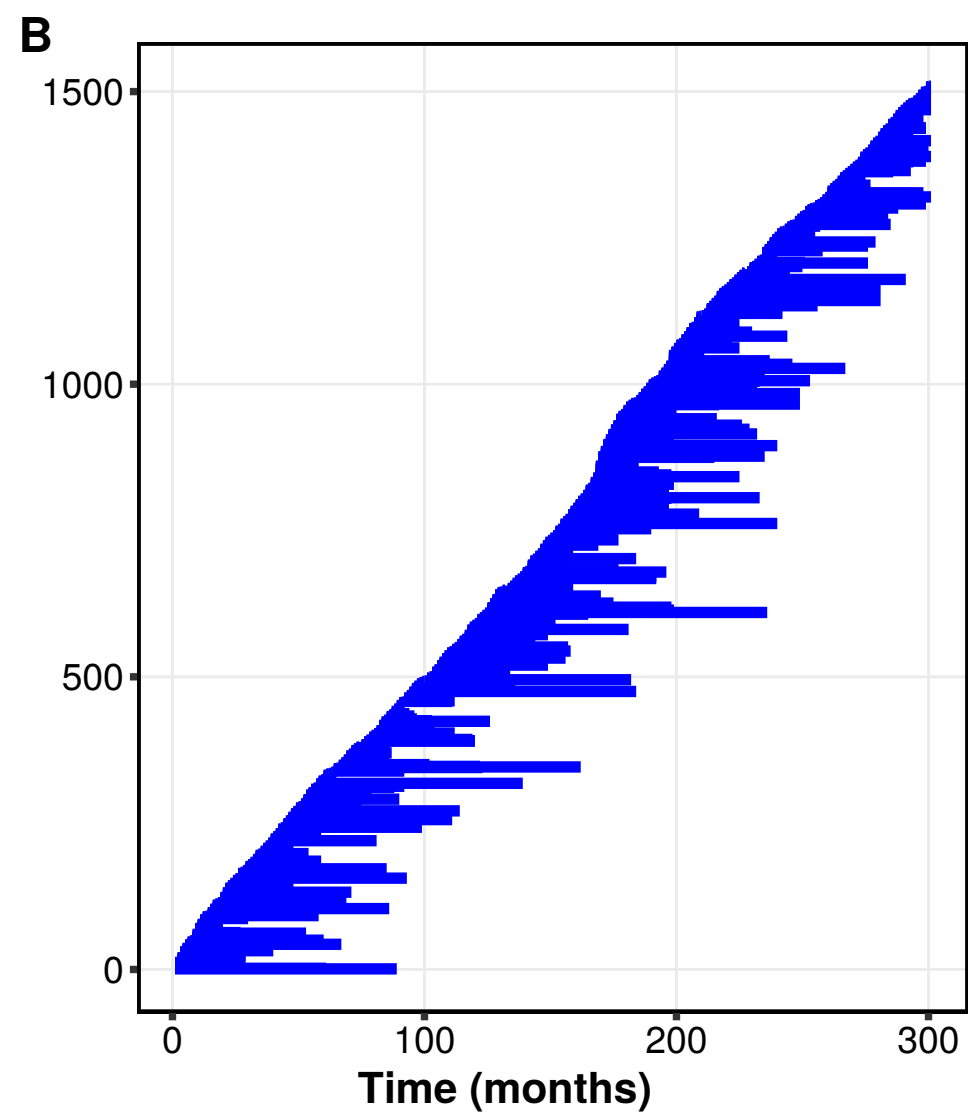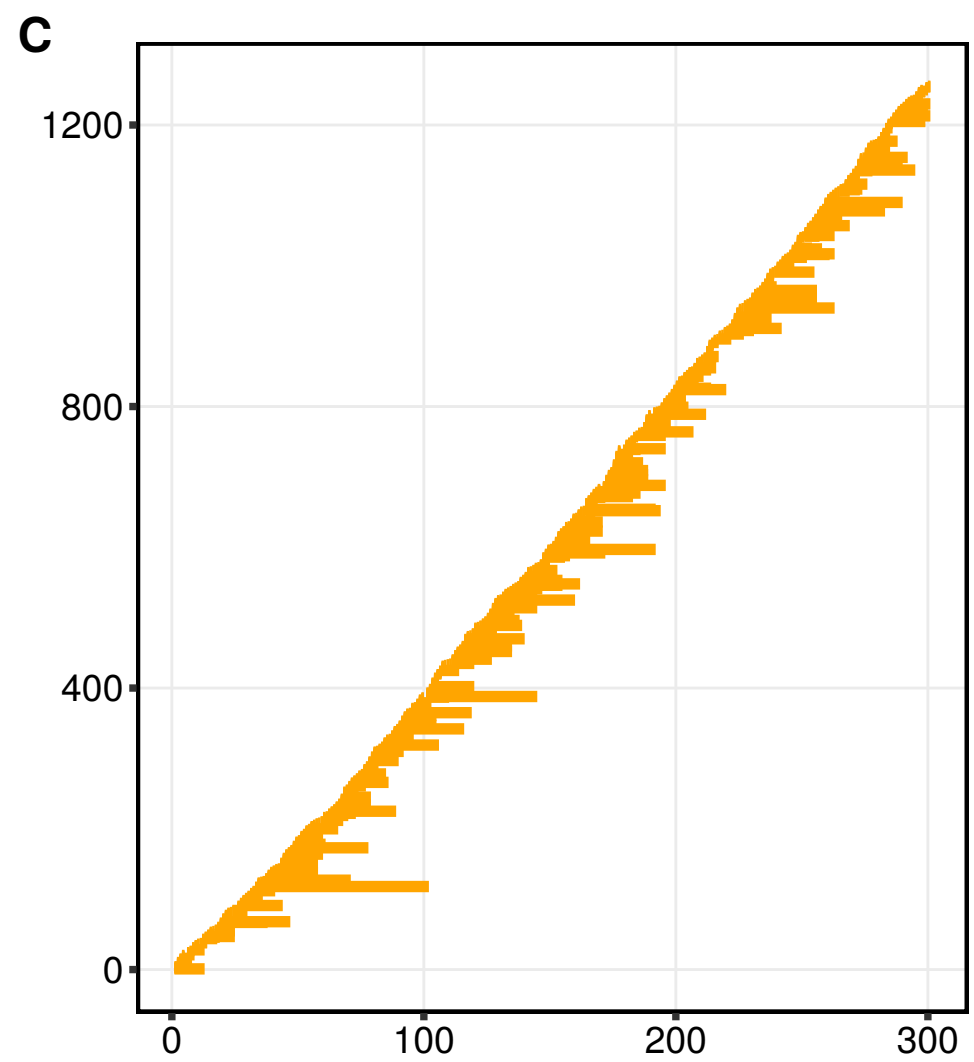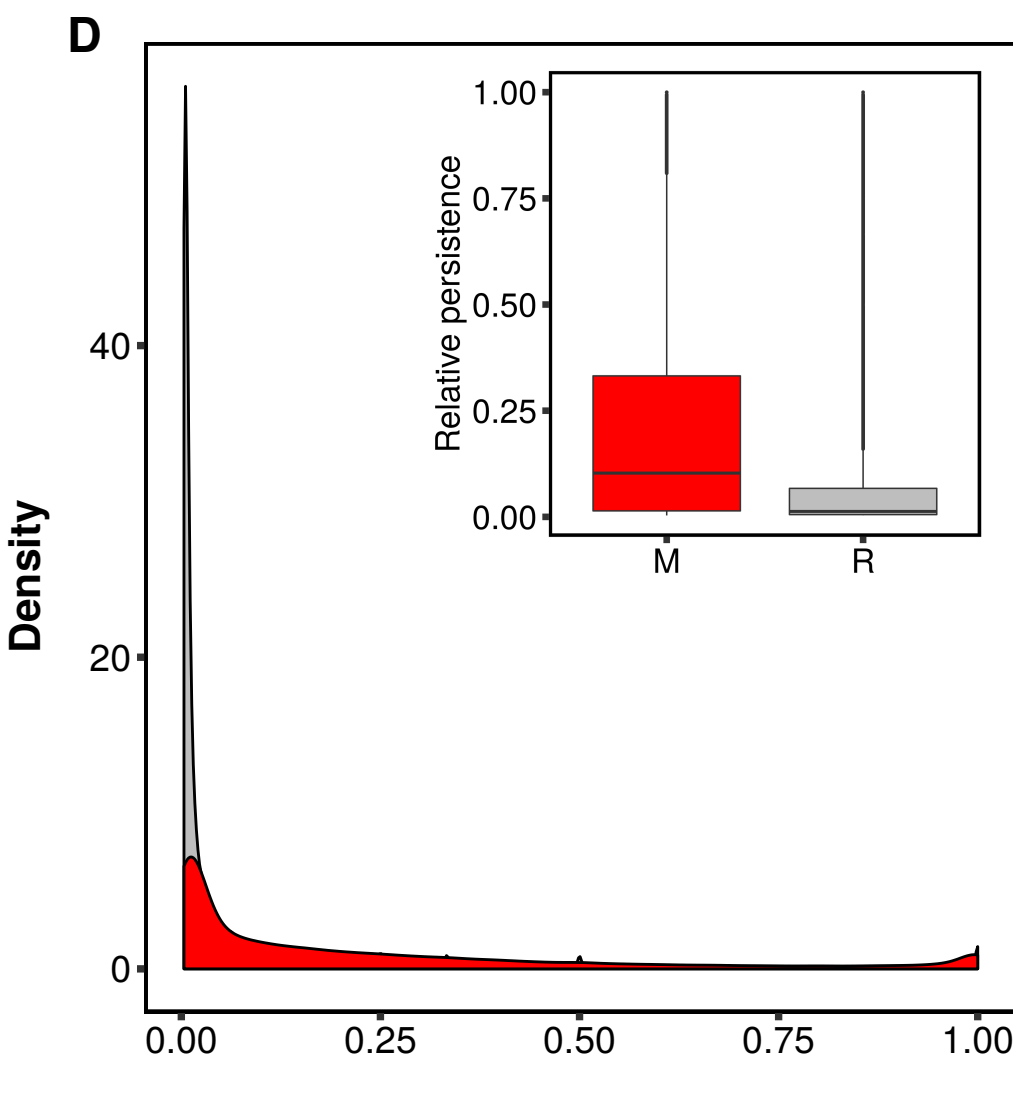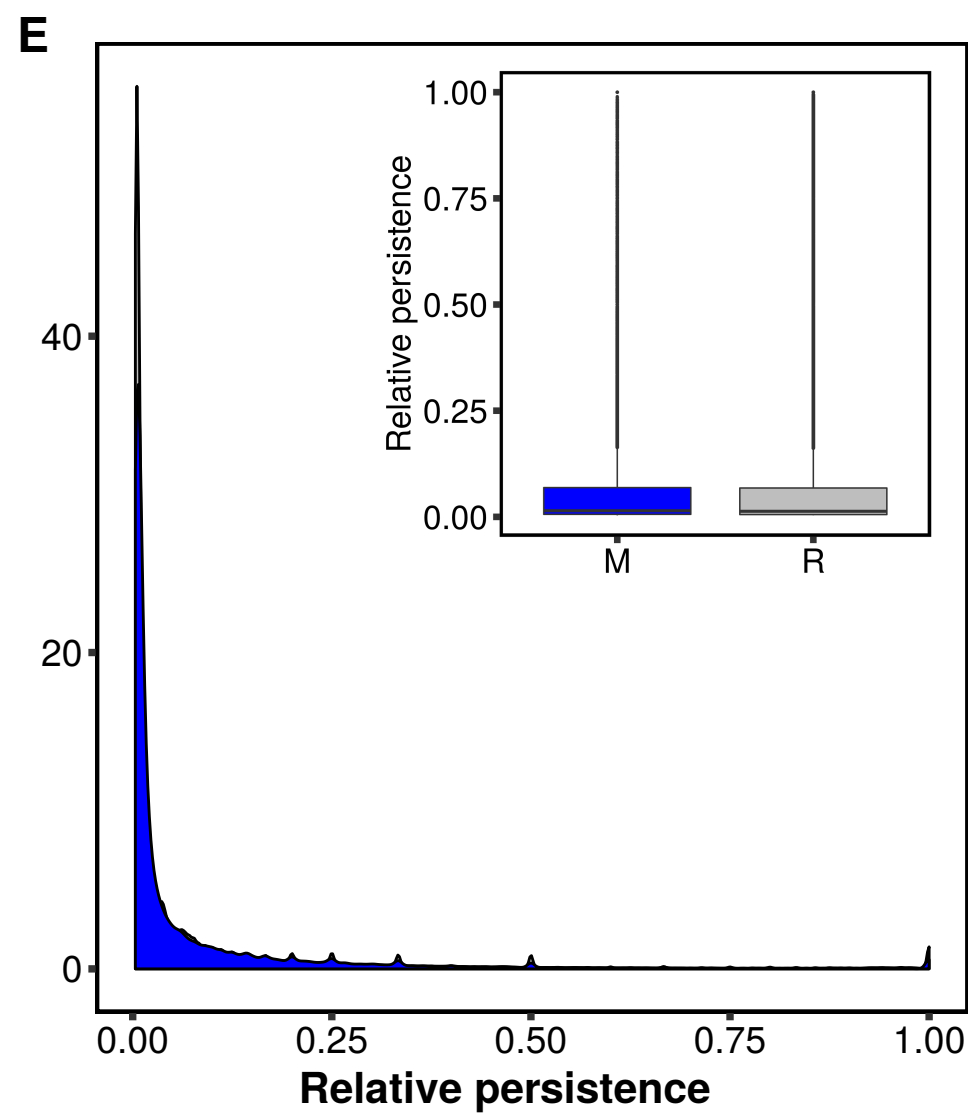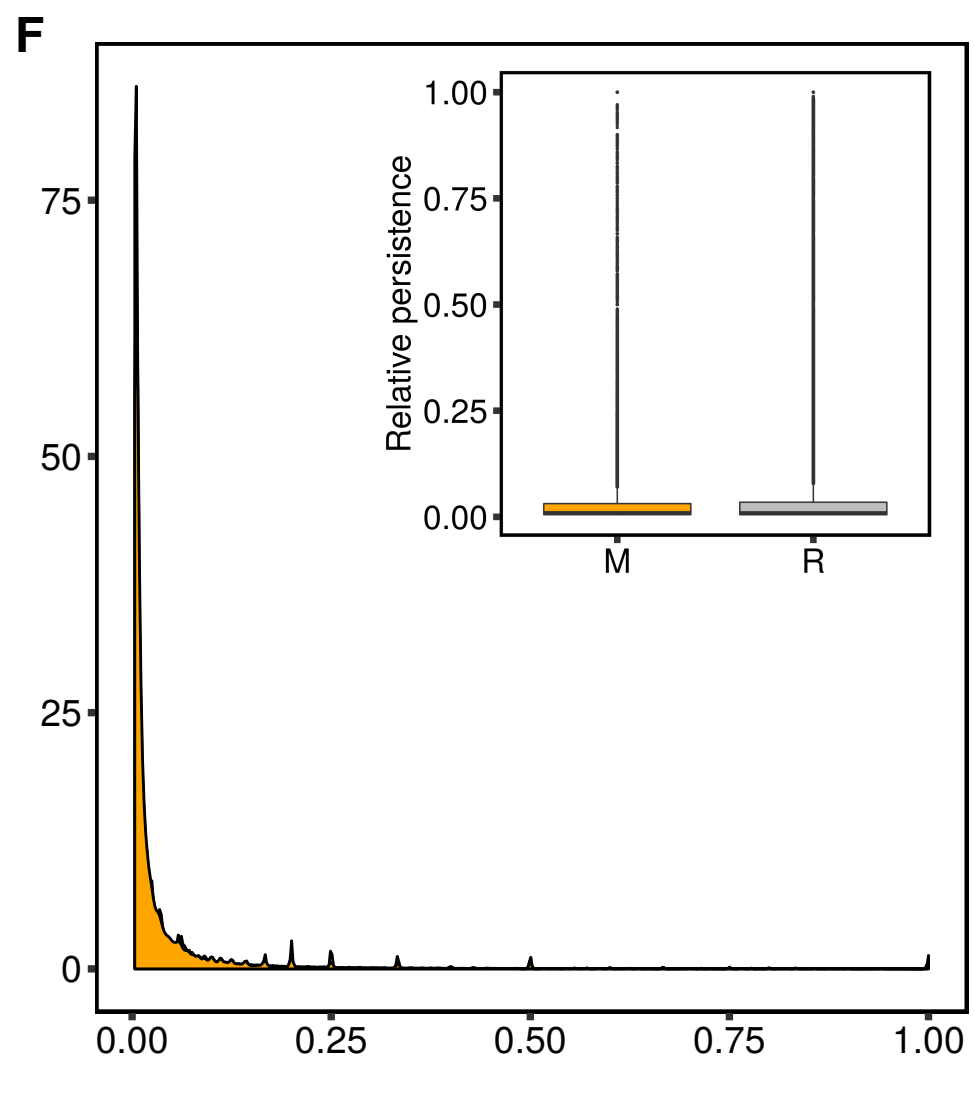

Supplement: S10 Fig — The left, middle, and right columns represent the selection (red), generalized immunity (blue), and neutral (orange) scenarios, respectively. (A,B,C) Example of representative population structure from one run of the ABM. Each line on the y-axis corresponds to a different module, and its horizontal length depicts its occurrence across layers. Modules are generated and die out. (D) The selection scenario is characterized by modules (red) that persist for much longer than the repertoires (gray) that compose them. The inset is a comparison between the values in the red and gray density plots using box plots. (E,F) In the neutral scenarios, modules and repertoires have similar relative persistence. See Methods for details on how we implemented seasonality and calculated relative persistence. Results in (D)–(E) are for data pooled across 50 runs of the ABM. Data from model output used to produce the figure can be found in https://figshare.com/articles/Figure_data/8079713. ABM, agent-based model. (PDF) [file pbio.3000336.s011.pdf]

Proportion of modules

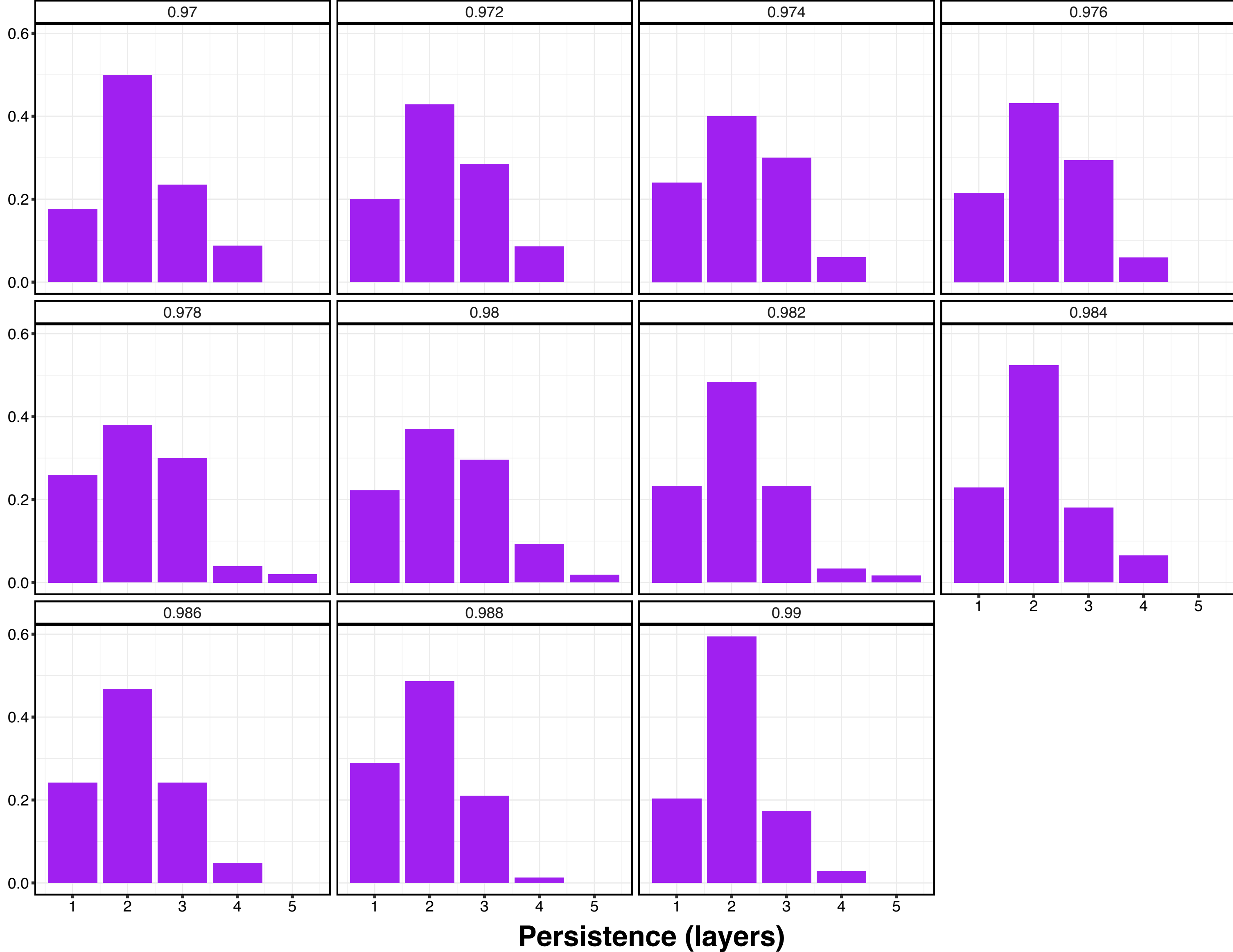

Supplement: S11 Fig — Because empirical networks are not necessarily identical to those produced in our theoretical work (for example, in size and genetic diversity), we explored the effect of thresholding. It is clear from the figure that cutoff does not have a major effect on the distribution of module persistence. We chose 97.8% because this was the point of transition at which modules started to persist for 5 layers. Data used to produce the figure can be found in https://figshare.com/articles/Figure_data/8079713. (PDF) [file pbio.3000336.s012.pdf]

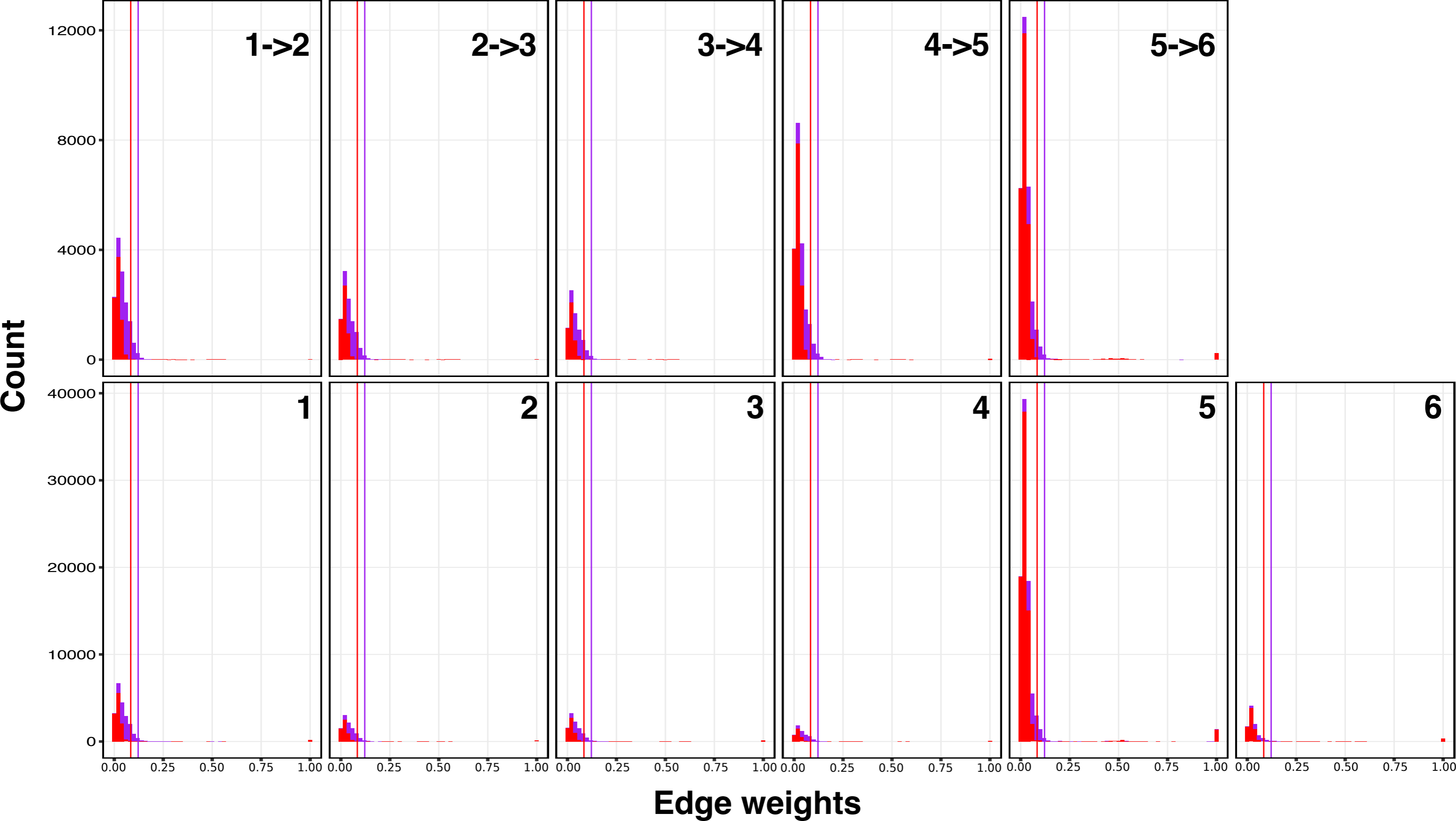

Supplement: S12 Fig — We assess how well our simulations for the immune selection scenario (red) matched the empirical data (purple) by comparing the distributions of edge weights. The lower and upper panels depict the distributions for the intra- and interlayer edges, respectively. Vertical lines represent the values where a cutoff of 97.8% was applied. Data from model output used to produce the figure can be found in https://figshare.com/articles/Figure_data/8079713. (PDF) [file pbio.3000336.s013.pdf]

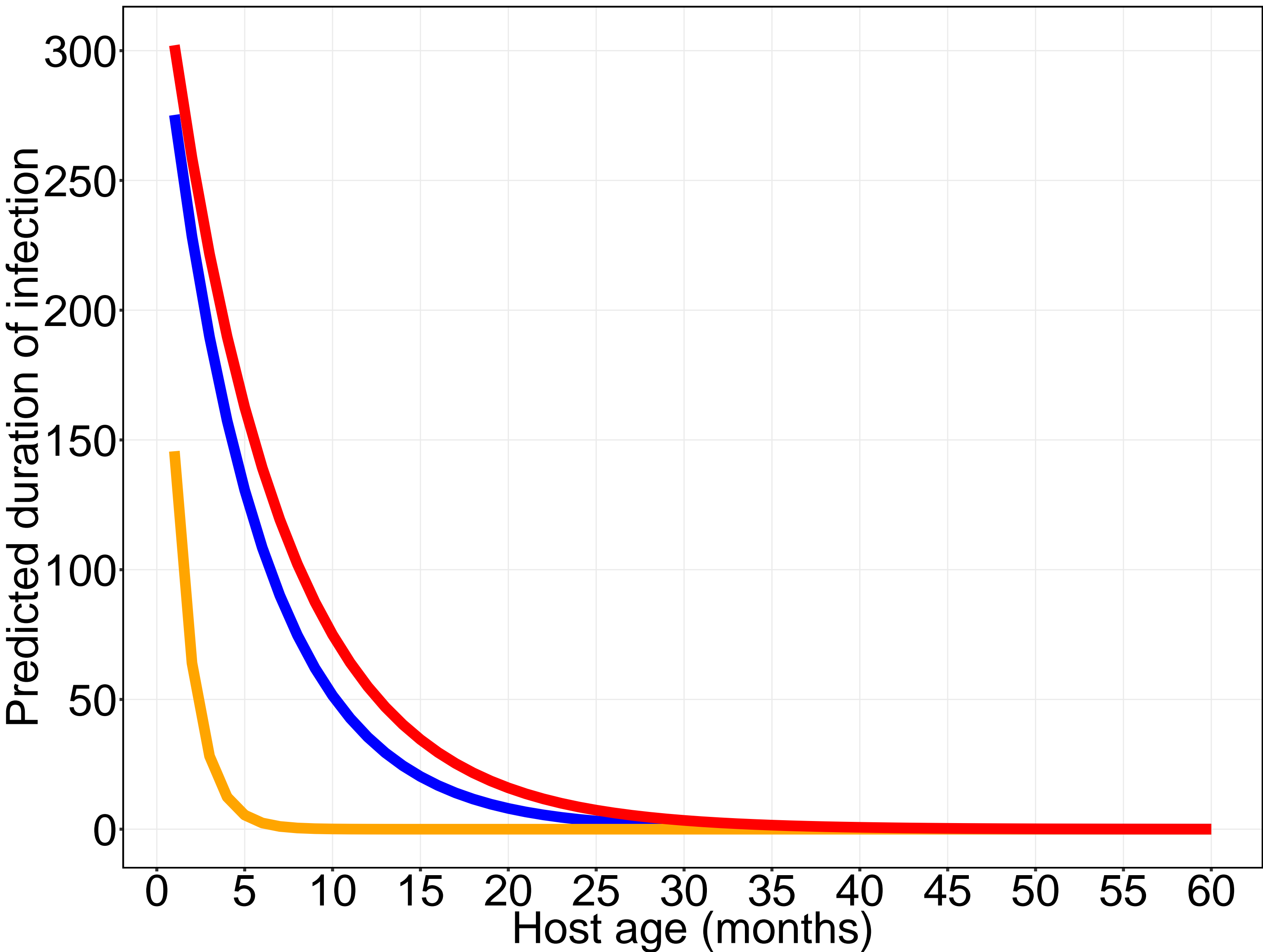

Supplement: S13 Fig — The lines depict the predicted values for duration of infection as a function of host age. The values were obtained for the exponential model d=ae−bi (where d is the duration of infection and i is the number of previous infections) that was fitted to the data from Fig 4D in the Main text. We used the EIR of each scenario and the number of accumulated infections to calculate host age. Curves of the standard error overlap with the curves of predicted values and are not presented (the standard error of model coefficient is very small). Red: Immune selection; blue: generalized immunity; orange: complete neutrality. Data used to produce the figure can be found in https://figshare.com/articles/Figure_data/8079713. EIR, entomological inoculation rate. (PDF) [file pbio.3000336.s014.pdf]
