# Supplementary material for: Competition for hosts modulates vast antigenic diversity to generate persistent strain structure in Plasmodium falciparum
Source: PLoS Biol. 2019 Jun 24;17(6):e3000336. doi: 10.1371/journal.pbio.3000336 (PMC6611651; doi:10.1371/journal.pbio.3000336)
Supplement: S1 Table — Extended details are in [15, 29]. Isolates are the number of people in which the parasite was detected by microscopy. Unique var types is the total number of genes obtained from the DBLα marker with the standard 96% similarity cutoff. Alleles is the number of alleles from the UpsBC var gene group that were detected in isolates with a single infection (MOI1; defined as isolates from which 40–55 var UpsBC genes were sampled). The last two columns describe the data used in the empirical analysis in the main text, with MOI1 being the number of nodes. DBLα, Duffy binding-like-alpha; MOI, multiplicity of infection; ups, upstream promoter. (PDF) [file pbio.3000336.s015.pdf]

Table S1. Summary of empirical data relevant for this study (extended details are in [1, 2]). Isolates are the number of people in which the parasite was detected by microscopy. **Unique *var* types** is the total number of genes obtained from the DBLα marker with the standard 96% similarity cutoff. **Alleles** is the number of alleles from the UpsBC *var* gene group that were detected in isolates with a single infection (**MOI1**; defined as isolates from which 40-55 var UpsBC genes were sampled). The last two columns describe the data used in the empirical analysis in the main text, with MOI1 being the number of nodes.

Survey	Sampling date	People sampled	Isolates	Unique *var* types	Alleles	MOI1
Sv1	Oct. 2012	1923	808	35,345	2866	90
Sv2	May-Jun. 2013	1902	513	27,586	2540	68
Sv3	May-Jun. 2014	1822	535	24,962	2505	69
Sv4	Oct. 2014	1866	430	16,222	2143	52
Sv5	Oct. 2015	2022	545	19,630	3019	115
Sv6	May-Jun. 2016	2091	272	18,103	2032	44

References

[1] Tiedje KE, Oduro A, Agongo G, Anyorigiya T, Azongo D, Awine T, et al. Seasonal Variation in the Epidemiology of Asymptomatic Plasmodium falciparum Infections Across Two Catchment Areas in Bongo District, Ghana. Am J Trop Med Hyg. 2017;97(1):199–212. doi:10.4269/ajtmh.16-0959.

[2] He Q, Pilosof S, Tiedje KE, Ruybal-Pesántez S, Artzy-Randrup Y, Baskerville EB, et al. Networks of genetic similarity reveal non-neutral processes shape strain structure in Plasmodium falciparum. Nat Commun. 2018;9(1):1817. doi:10.1038/s41467-018-04219-3.
